# *Akkermansia muciniphila* uses human milk oligosaccharides to thrive in the early life conditions in vitro

**DOI:** 10.1038/s41598-020-71113-8

**Published:** 2020-08-31

**Authors:** Ioannis Kostopoulos, Janneke Elzinga, Noora Ottman, Jay T. Klievink, Bernadet Blijenberg, Steven Aalvink, Sjef Boeren, Marko Mank, Jan Knol, Willem M. de Vos, Clara Belzer

**Affiliations:** 1 Laboratory of Microbiology, Wageningen University, Stippeneng 4, 6708 WE Wageningen, The Netherlands; 2 Laboratory of Biochemistry, Wageningen University, Stippeneng 4, 6708 WE Wageningen, The Netherlands; 3 Danone Nutricia Research, Uppsalalaan 12, 3584 CT Utrecht, The Netherlands; 4 Human Microbiome Research Program, Faculty of Medicine, University of Helsinki, P.O. Box 66, 0014 Helsinki, Finland

**Keywords:** Microbiome, Microbiology, Microbial ecology

## Abstract

*Akkermansia muciniphila* is a well-studied anaerobic bacterium specialized in mucus degradation and associated with human health. Because of the structural resemblance of mucus glycans and free human milk oligosaccharides (HMOs), we studied the ability of *A. muciniphila* to utilize human milk oligosaccharides. We found that *A. muciniphila* was able to grow on human milk and degrade HMOs. Analyses of the proteome of *A. muciniphila* indicated that key-glycan degrading enzymes were expressed when the bacterium was grown on human milk. Our results display the functionality of the key-glycan degrading enzymes (α-l-fucosidases, β-galactosidases, exo-α-sialidases and β-acetylhexosaminidases) to degrade the HMO-structures 2′-FL, LNT, lactose, and LNT2. The hydrolysation of the host-derived glycan structures allows *A. muciniphila* to promote syntrophy with other beneficial bacteria, contributing in that way to a microbial ecological network in the gut. Thus, the capacity of *A. muciniphila* to utilize human milk will enable its survival in the early life intestine and colonization of the mucosal layer in early life, warranting later life mucosal and metabolic health.

## Introduction

*Akkermansia muciniphila* is a Gram-negative anaerobe, belonging to the phylum Verrucomicrobia^1^, that colonizes the mucus layer of the human gastrointestinal (GI) tract^2^. *A. muciniphila* is associated with a healthy mucosal layer and metabolic state as it has been inversely correlated with obesity^3,4^, metabolic diseases (Type 2 diabetes)^5^ as well as intestinal disorders (inflammatory bowel disease (IBD) and appendicitis)^6–8^. This intestinal bacterium has an extraordinary capacity to degrade mucin as the sole energy, carbon and nitrogen source and convert this polymer into mostly acetate and propionate^2^.

*Akkermansia muciniphila *is a common member of the adult and infant microbiota^9^. The infant’s gut colonisation with *A. muciniphila* has been detected from the first month of life, with a continuously increasing abundance during adulthood^9,10^. Additionally, *A. muciniphila* was detected in the breast tissue of lactating mothers as well as in human milk^11–13^. Interestingly, two studies have reported that *A. muciniphila* was found to be lower in abundance in the breast-fed infants compared to formula-fed infants^14,15^. In a more recent study with 98 Swedish infants though, the abundance and the prevalence of *A. muciniphila* increased between 4 and 12 months old, and showed no significant change depending on delivery mode or type of feeding^16^. Early in life, mother’s milk is often the only source of nutrients and dietary glycans for infants, and it is considered the best nourishment for the development of the new-born^17^. The glycans in human milk are named human milk oligosaccharides (HMOs), and they have proven to have an impact on infant intestinal microbiota composition^18^. Human milk contains 5–15 g/l HMOs, with more than 200 different HMO structures reported of which 100 have been successfully elucidated^19–22^. The presence and the quantity of these HMOs structures vary per individual and are related to the genetic Secretor and Lewis status of the mother^23^. The major building blocks of monosaccharides present in HMOs are d-glucose (Glc), d-galactose (Gal), *N*-acetyl-glucosamine (GlcNAc), l-fucose (Fuc), and *N*-acetylneuraminic acid (sialic acid, Neu5Ac)^24^. These sugars form a number of complex glycans containing well-defined different glycosidic linkages resulting in both linear and branched structures^25^. In human milk 70% of the oligosaccharides are fucosylated and 30% are sialylated^19,26^. HMOs can function as prebiotic substrates by promoting and stimulating the growth of beneficial bacteria such as bifidobacteria^27^. Nowadays, supplementation of infant formulae with HMOs, such as 2′-FL and LNnT, is gaining more and more interest to bring infant formula composition even closer to human milk^28^. In addition, some of these oligosaccharide molecules inhibit the colonisation of pathogenic bacteria by acting as receptor analogues and binding to the bacterial surface^24^.

The resemblance of glycosidic structure between HMOs and mucin glycans might explain why some bacteria are capable of utilising both human milk glycans and host mucosal glycans (mucins)^29,30^. Mucins are the main structural components of the mucus layer that covers the gut epithelium surface. Mucins’ protein core consists of 80% carbohydrates, mainly *N-*acetylgalactosamine, *N-*acetylglucosamine, fucose, galactose and sialic acid^31,32^. The mucus layer in the human gut is divided into an outer layer, which provides a nutrient-rich habitat for the microbiota, and an inner layer, which is firmly attached to the surface of the epithelium and virtually free of bacteria^33^. In the human gut, *A. muciniphila* has the extraordinary capacity to degrade mucins by employing a large arsenal of sulfatases and glycoside hydrolases (GH) for effective metabolism of mucin glycans such as α-fucosidases, α-sialidases, β-galactosidases, β-acetylhexosaminidases, and α-acetylglucosaminidases^1,34^.

We hypothesize that the presence of *A. muciniphila* in the early life intestine is the result of its ability to use its mucin degrading machinery to breakdown HMOs. Its presence in the early life intestine will enhance microbial ecologic network formation and healthy microbial colonisation of the mucosal layer warranting later life health. To assess this, we tested the ability of *A. muciniphila* to grow on human milk and different HMOs. Subsequently, we identified the HMOs structures that *A. muciniphila* was able to break down and the enzymes responsible for the degradation.

## Results

### *A. muciniphila* grows on human milk via HMOs utilisation

**Figure 1:**
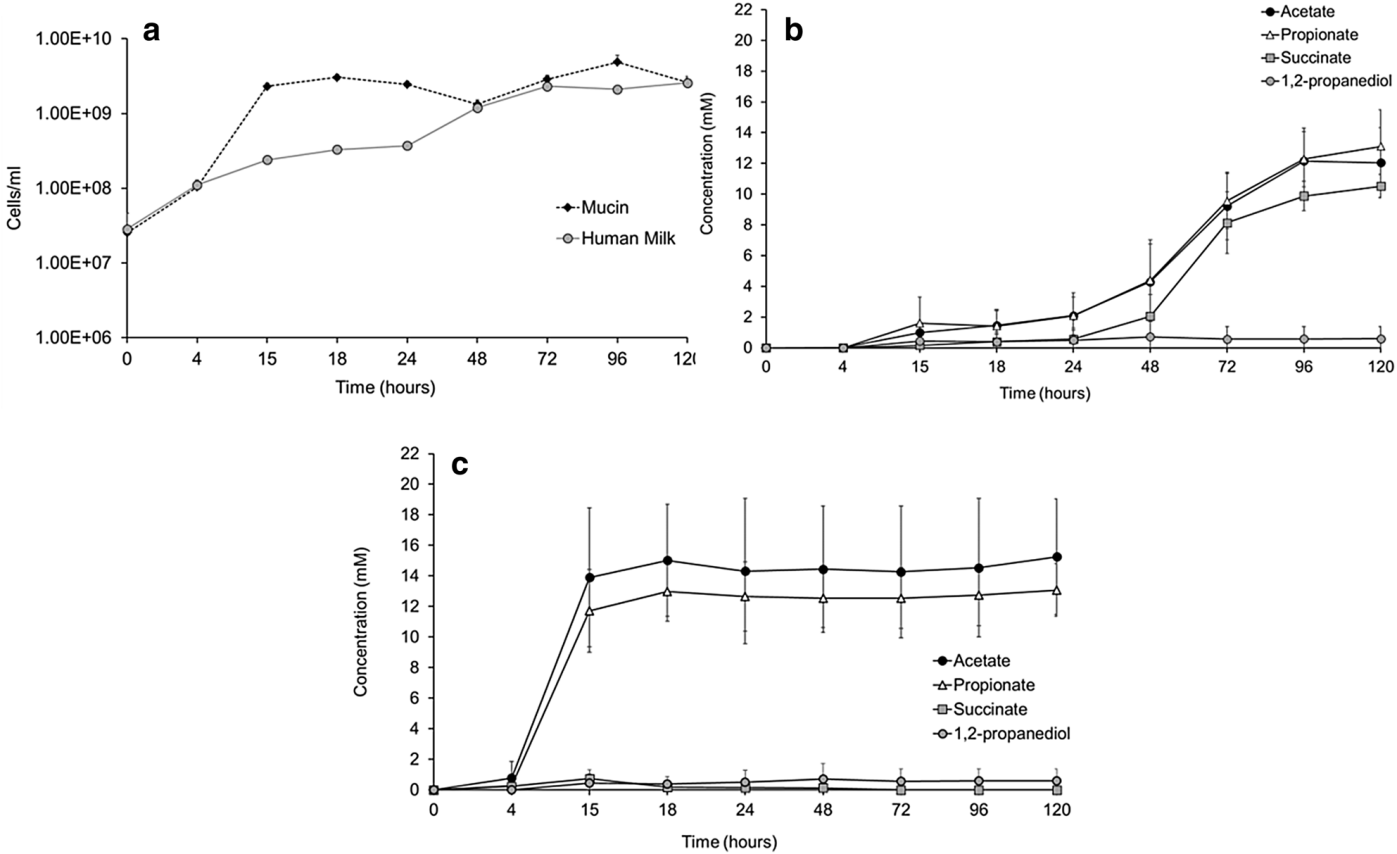
*A. muciniphila* growth in human milk. (**a**) *A. muciniphila* growth in human milk or porcine mucin as the sole carbon and nitrogen source. Error bars indicate the standard error of qPCR for three biological replicates. *A. muciniphila* SCFAs production (**b**) in human milk and (**c**) in mucin. Error bars indicate the standard deviation of three biological replicates.

**Figure 2:**
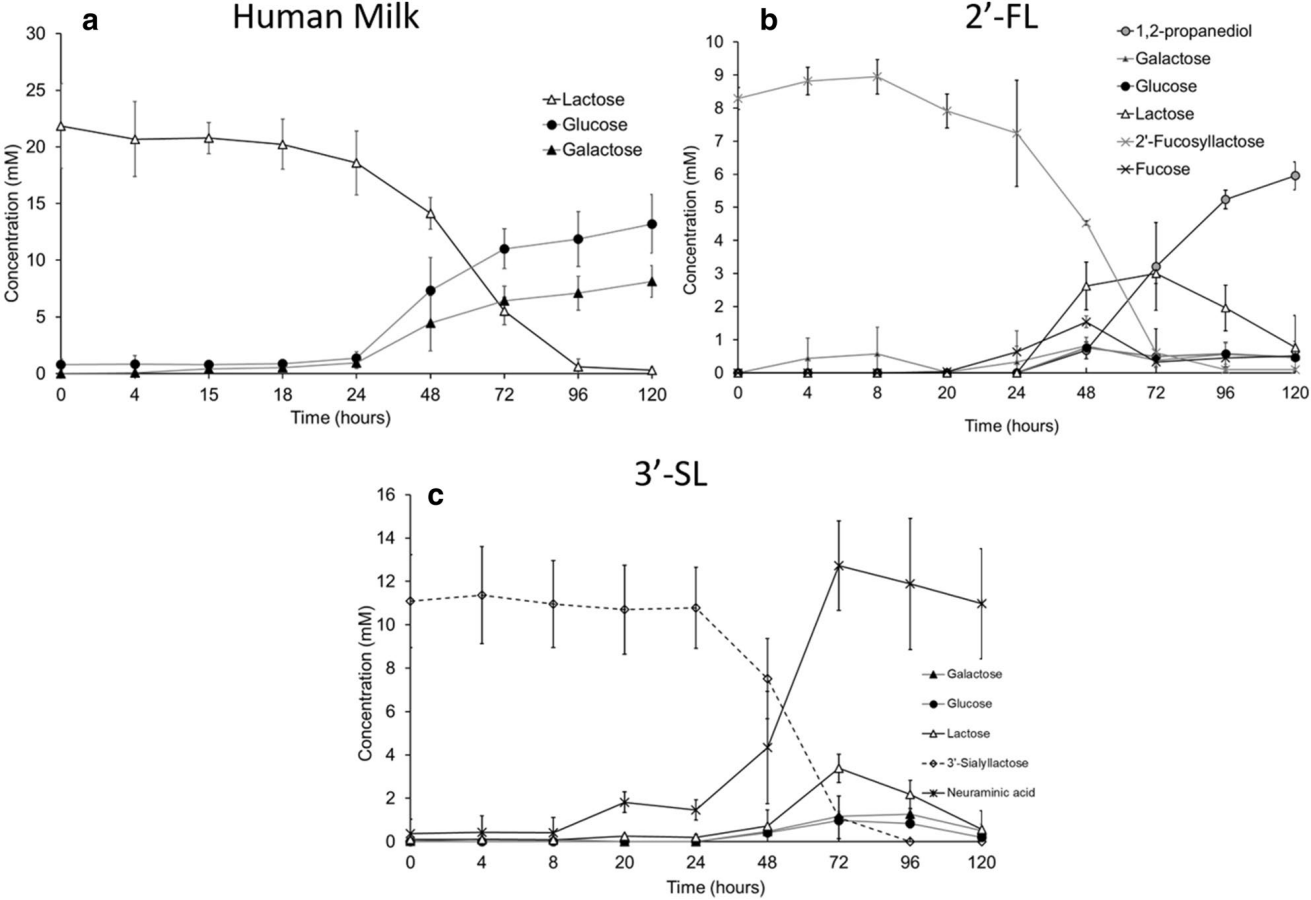
*A. muciniphila* HMO degradation. (**a**) Human Milk, (**b**) 2′-fucosyllactose (2′-FL), and (**c**) 3′-sialyllactose (3′-SL). Error bars represent the standard deviation of three biological replicates.

**Figure 3:**
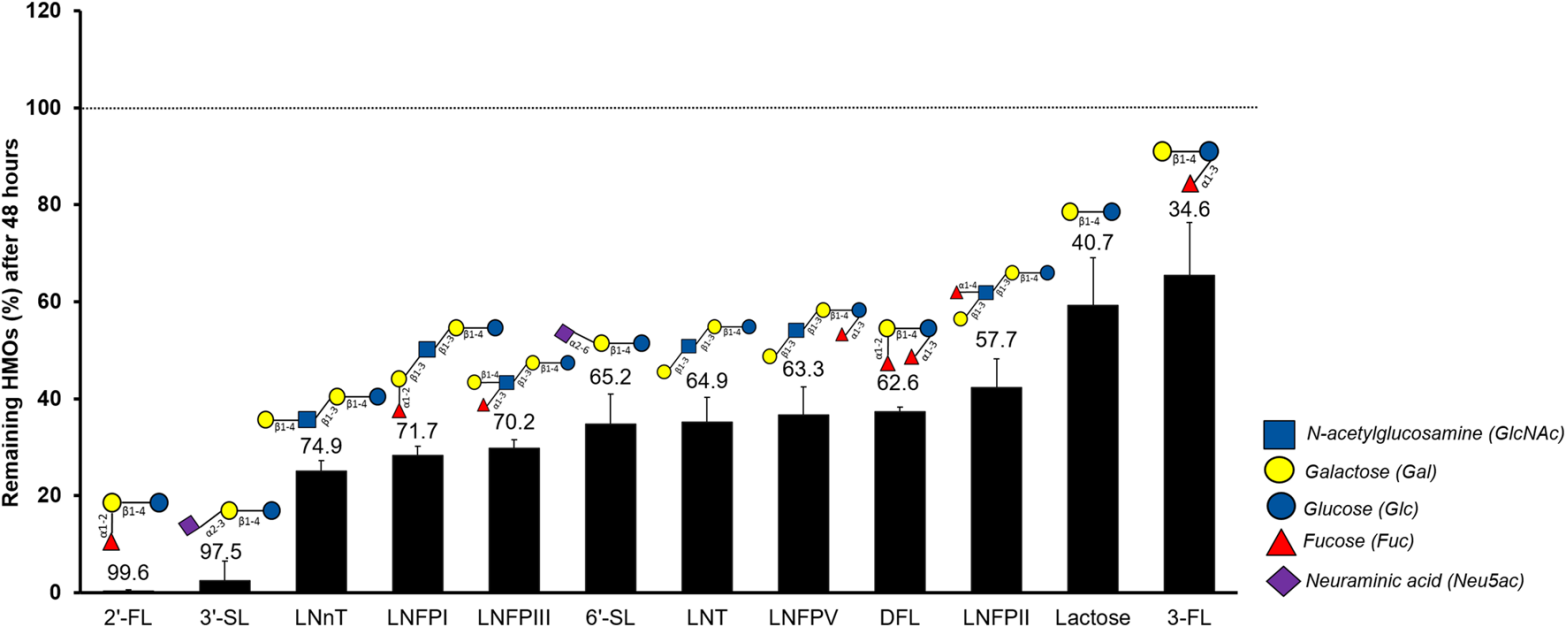
Utilisation of HMOs structures by *A. muciniphila* incubated in 10% human milk. The numbers above each bar indicate the degradation percentage of each HMO. Error bars represent the error propagation of three biological replicates.

Incubation of *A. muciniphila* on human milk resulted in growth (Fig. 1a). The fermentation profile of the cultures showed production of acetate, propionate, and succinate (Fig. 1b,c) as well as release of glucose and galactose due to the utilisation of the lactose that is already present in the breast milk and in the HMOs (Fig. 2a). The amounts of produced short-chain fatty acids (SCFAs) and utilised sugars are shown in the Supplementary Table 2. We next sought to investigate which HMOs structures were utilised by *A. muciniphila* during the growth on human milk. The HMOs profile showed utilisation of neutral trioses (2′-fucosyllactose [2′-FL] and 3-fucosyllactose [3-FL]), tetraoses (difucosyllactose [DFL], lacto-*N*-tetraose [LNT], lacto-*N*-neotetraose [LNnT]), pentaoses (lacto-fucopentaose I [LNFP I], lacto-fucopentaose II [LNFP II], lacto-fucopentaose III [LNFP III], lacto-fucopentaose V [LNFP V]), and acidic trioses (3′-siallylactose [3′-SL], 6′-sialyllactose [6′-SL]) (Fig. 3). All measured HMOs were reduced at least two-fold (Fig. 3). The HMO profile analysis revealed that *A. muciniphila* utilises almost completely 2′-FL and 3′-SL present in human milk (99.65% and 97.49%, respectively) (Fig. 3).

Next, it was tested whether these nutritional components by themselves could promote the growth of *A. muciniphila*. Incubation of *A. muciniphila* with pure 2′-FL or 3′-SL (10 mM) resulted in growth (Supplementary Fig. 1) and demonstrated the release of lactose from both 2′-FL or 3′-SL (Fig. 2b,c). The resulting lactose was further broken down to its monosaccharides (glucose and galactose). Growth on 2′-FL resulted in the production of 1,2-propanediol (5.95 ± 0.42 mM), which is an indication of the fucose metabolism by *A. muciniphila*. The liberated neuraminic acid from 3′-SL was not further catabolised by the bacterium. The overall fermentation efficiency was determined by calculating the carbon balance; the recovery of carbon atoms at 48 h of 2′-FL and 3′-SL was 86.58 and 82.58% respectively (Supplementary Table 3a). Furthermore, the amounts of produced short-chain fatty acids (SCFAs) and utilised sugars of *A. muciniphila* grown in 2′-FL and 3′-SL are shown in the Supplementary Table 3b.

### *A. muciniphila* exhibits glycoside hydrolase expression during growth on human milk

We performed proteomic analysis on the *A. muciniphila* cultures grown on human milk to identify the active enzymes that could contribute to the degradation of human milk and its components. A total of 832 bacterial proteins were detected after growth on human milk. We mined the proteome data for proteins predicted to participate in carbohydrate metabolism and this returned 109 proteins and over half of them (62 proteins) are primarily involved in this specific metabolic pathway (Table 1). *A. muciniphila* possesses 58 proteins that encode for glycoside hydrolases (GHs). In our results, we detected 43 GHs and 19 of these proteins belong to the six GH families (GH2, GH20, GH29, GH33, GH35 and GH95) that target the most common linkages found within HMOs. We identified four β-galactosidases that belong to GH2 family, two GH35 β-galactosidases, seven β-hexosaminidases from GH family 20, two GH29 α-fucosidases, two GH95 α-fucosidases and two exo-alpha sialidases from GH33 (Table 1). Therefore, we proposed *A. muciniphila* employed the fucosidases to release the terminal α1-2/3/4 linked fucose from the fucosylated HMOs; 2′-FL (Fucα1-2Galβ1-4Glc), 3-FL (Galβ1-4Fucα1-3Glc), DFL (Fucα1-2Galβ1-4 Fucα1-3Glc), LNFPI (Fucα1-2Galβ1-3GlcNAcβ1-3Galβ1-4Glc), LNFP II (Galβ1-3Fucα1-4GlcNAcβ1-3Galβ1-4Glc), LNFP III (Galβ1-4Fucα1-3GlcNAcβ1-3Galβ1-4Glc) and LNFP V (Galβ1-3GlcNAcβ1-3Galβ1-4Fucα1-3Glc). Additionally, *A. muciniphila* expressed the 2 sialidases that can liberate the α2-3 and α2-6 linked sialic acid from the sialylated glycans 3′-SL (Neu5Acα2-3Galβ1-4Glc) and 6′-SL (Neu5Acα2-6Galβ1-4Glc) present in the human milk. Furthermore, the hydrolysis of the terminal β1-3 and β1-4 galactose residues found in the HMOs structures; LNT (Galβ1-3GlcNAcβ1-3Galβ1-4Glc), LNnT (Galβ1-4GlcNAcβ1-3Galβ1-4Glc), lactose (Galβ1-4Glc), as well as LNFP II, LNFP III and LNFP V can be the result of β-galactosidases’ action expressed by *A. muciniphila* during growth on human milk. *A. muciniphila* expressed also 7 β-hexosaminidases which are responsible for releasing the terminal β1-3 and β1-4*N-*acetylglucosamine (GlcNAc) bound to glycan structures. However, GlcNAc is usually found as terminal sugar in HMO glycans but rather is often decorated by other monosaccharides (galactose, fucose, sialic acid). Thus, *A. muciniphila* might use its hexosaminidases to degrade simpler HMOs such as Lacto-*N*-biose (Galβ1-3GlcNAc), LNT2 (GlcNAcβ1-3Galβ1-4Glc). In addition, *A. muciniphila* expressed some of the necessary enzymes to metabolise the liberated monosaccharides from the HMOs degradation. *A. muciniphila* employs, for example, Amuc_1833 (l-fucose transporter-fucP), Amuc_1832 (l-fucose isomerase-fucI), Amuc_1830 (l-fuculokinase-fucK), and Amuc_1829 (class II aldolase-fucA) for the utilisation of the liberated fucose to 1,2-propanediol. Furthermore, Amuc_0969 (Galactokinase) participates in the first steps of the galactose metabolism by converting the free galactose into α-d-galactose-1-phosphate, while Amuc_0097 (ROK family protein) can be used by *A. muciniphila* to convert glucose to α-d-glucose-6-phosphate during the first step of the glycolysis pathway (Supplementary Table 4). Nevertheless, *A. muciniphila* lacks all the necessary enzymes for the sialic acid utilisation, since no expression of these enzymes was observed in the proteome data. These data demonstrate that *A. muciniphila* has the enzymatic capacity to utilise a broad range of HMOs as well as their constituents.

**Table 1:**
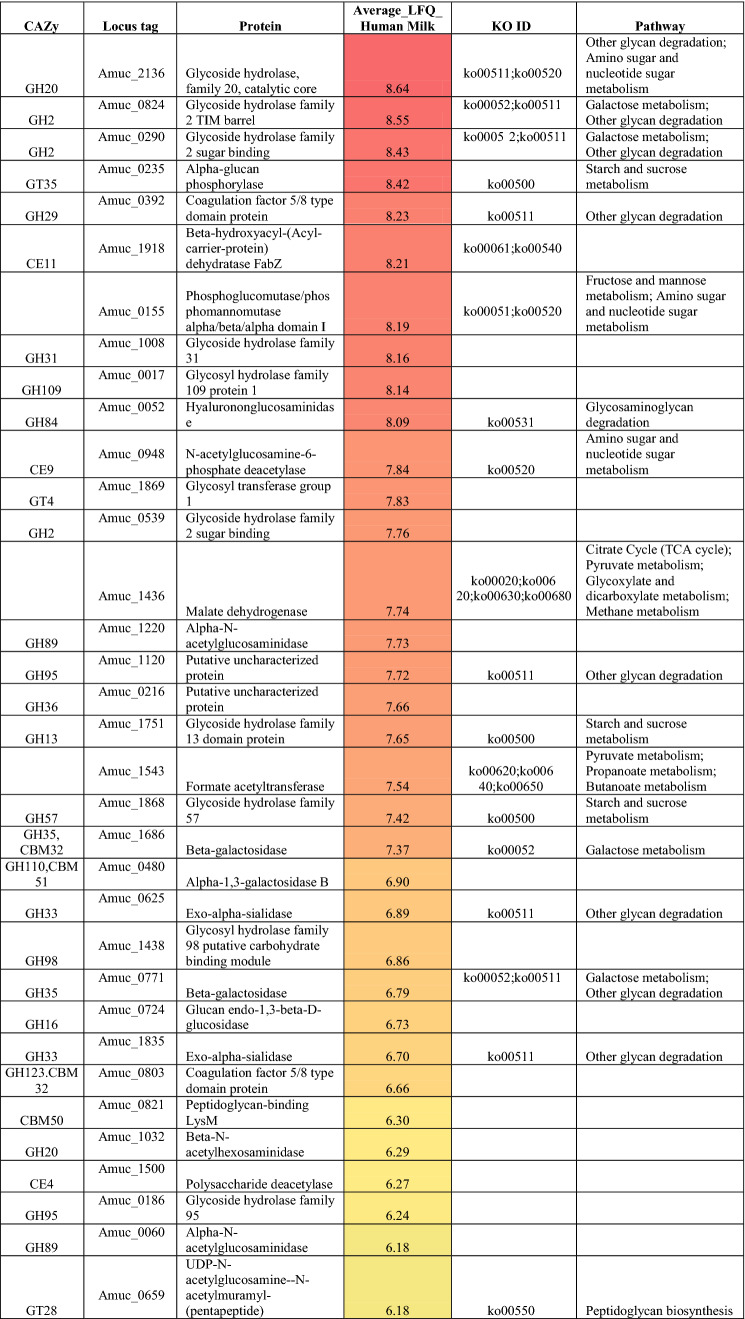

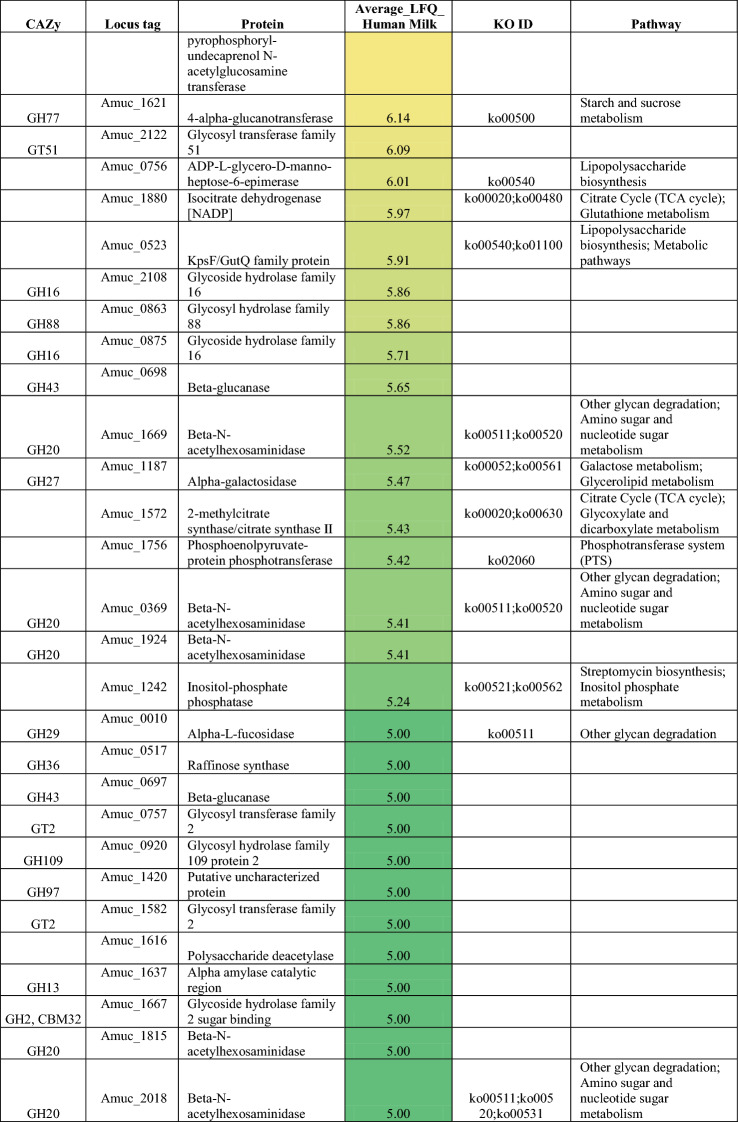
Abundance of *A. muciniphila* enzymes involved in carbohydrate metabolism with their corresponding KEGG identifier (KO ID).

The average of Log10 transformed LFQ values is shown. Colouring is based on abundance from the most abundant (red) to medium abundant (orange) to least abundant (green).

### Characterization of *A. muciniphila* HMO degrading enzymes

The next step of the study was concerned with the characterisation of *A. muciniphila* enzymes that are effective in HMO-degradation. Therefore, we assessed five glycan-degrading enzymes identified by proteomics that are also predicted to hydrolyse the glycosidic bonds of lactose and the HMOs; 2′-FL, LNT and LNT2 (Table 1); one α-fucosidase (Amuc_0010), two β-galactosidases (Amuc_0771 and Amuc_1686) and two β-acetylhexosaminidases (Amuc_0369 and Amuc_2136). The enzymatic activity of the recombinant proteins was first assessed using synthetic substrates (Table 2). Amuc_0771 and Amuc_2136 showed a pH optimum of 5.0, while Amuc_0010 and Amuc_0369 displayed optimal pH at 5.6. For Amuc_1686 the rate of pNP release (μΜ min^−1^) was similar for all the different pH values that were tested (Supplementary Fig. 2). pH 5.0 was selected as the optimal pH for this enzyme. Each enzyme’s optimal pH and temperature of 37 °C were used to assess the kinetic parameters of these proteins against synthetic substrates pNP-α-l-Fuc, pNPG and GlcNAc-β-pNP (Table 2 and Supplementary Fig. 3). The α-l-fucosidase (Amuc_0010) showed a *K*_M_ 839 ± 46.72 μM against pNP-Fucose. Both β-acetylhexosaminidases (Amuc_0369 and Amuc_2136) exhibited hexosaminidase activity by cleaving GlcNAc from pNP-GlcNAc. A lower *K*_M_ for Amuc_0369 (322.37 ± 43.64 μM) was observed than Amuc_2136 (714.55 ± 47.96 μM). Cleavage of galactose from pNPG was detected with β-galactosidases (Amuc_0771 and Amuc_1686). Amuc_0771 showed higher *K*_M_ (2,599 ± 565.27 μΜ) than Amuc_1686 (319.40 ± 259.30 μΜ). The ability of the α-l-fucosidase (Amuc_0100) for cleaving α1-2 linked fucose from 2′-FL (Fucα1-2Galβ1-4Glc) was assessed (Table 2 and Supplementary Fig. 4). The enzyme exhibited a high *K*_M_ and lower activity (152.08 min^−1^ *k*_cat_) against 2′-FL than pNP-Fuc (2.27 × 10^4^ min^−1^ *k*_cat_)*.* The activity of β-acetylhexosaminidases (Amuc_0369 and Amuc_2136) on the HMOs, LNT (Galβ1-3GlcNAcβ1-3Galβ1-4Glc) and LNT2 (GlcNAcβ1-3Galβ1-4Glc) was assessed as well. The substrate and product specificity were monitored by HPAEC-PAD. For both enzymes no liberation of GlcNAc was observed in the presence of LNT, while both *A. muciniphila* β-acetylhexosaminidases were able to cleave the terminal GlcNAc off lactose resulting in LNT2. Amuc_0369 exhibited a *k*_cat_ 1.42 × 10^4^ min^−1^ and a *K*_M_ 3,980 ± 210.30 μM against LNT2. Amuc_2136 showed higher substrate affinity than Amuc_0369 (2,435.82 ± 289.51 μM *K*_M_) but no significant difference in the catalytic activity (1.45 × 10^4^ min^−1^ *k*_cat_). The β-galactosidases (Amuc_0771 and Amuc_1686) were tested for their capability to break down galactosidic linkages from the HMO, LNT (Galβ1-3GlcNAcβ1-3Galβ1-4Glc) and lactose (Galβ1-4Glc). Amuc_0771 showed a *k*_cat_ 4.42 × 10^3^ min^−1^ and a *K*_M_ 1,223 ± 171.7 μM against LNT (β1-3) (Table 2). In addition, Amuc_0771 was able to release 711 μΜ of glucose and 936 μΜ of galactose when was incubated in 2,000 μΜ of lactose overnight (Supplementary Fig. 5). However, the reaction in lactose was slow and therefore we were not able to assess the kinetics of Amuc_0771. The enzyme displayed cleaving capacity for both β1-3 and β1-4 glycosidic linkages, but with higher substrate specificity towards β1-3 linkages. The other β-galactosidase (Amuc_1686) showed no enzymatic activity against LNT or lactose. *E. coli* BL21 Rosetta strain harbouring the pCDF1-b empty vector induced with IPTG was used as negative control and it was incubated overnight in 2 mM LNT, 2′-FL, lactose and 5 mM LNT2 showing no activity at all, against these substrates (Supplementary Fig. 7).

**Table 2:** Kinetic parameters of α-L-fucosidase (Amuc_0010), β-hexosaminidases (Amuc_0369 and Amuc_2136) and β-galactosidases (Amuc_0771 and Amuc_1686) with synthetic substrates and HMOS.

	Substrate	Vmax (μM min^−1^)	k_cat_ (min^−1^)	K_M_ (μΜ)	k_cat_/K_M_ (min^−1^ μM^−1^)
**a-L-fucosidase**	**Amuc_0010**				
	PNP-Fucose	19.76 ± 11.30	2.27E + 04	841.23 ± 46.72	27.07
	2′-Fucosyllactose	25.13 ± 6.80	152.08	1.03 × 10^4^ ± 6.98 × 10^3^	0.02
**β-hexosaminidases**	**Amuc_0369**				
	PNP-GlcNAc	55.64 ± 2.02	4.02 × 10^4^	323.38 ± 43.64	124.50
	Lacto-*N*-triose II	982.61 ± 37.68	1.42 × 10^4^	3,980 ± 210.30	3.56
	**Amuc_2136**				
	PNP-GlcNAc	109.46 ± 4.22	8.93 × 10^4^	714.55 ± 47.96	125.10
	Lacto-*N*-triose II	443.75 ± 47.20	1.45 × 10^4^	2,435.82 ± 289.51	5.95
**β-galactosidases**	**Amuc_0771**				
	PNP-Galactose	60.08 ± 7.02	825.63	2,599 ± 565.27	0.34
	Lacto-*N*-tetraose	302.08 ± 53.71	4.42 × 10^3^	1,223 ± 171.70	3.61
	**Amuc_1686**				
	PNP-Galactose	29.34 ± 3.80	1.63 × 10^3^	319.40 ± 259.30	4.21

### *A. muciniphila* expresses mucus-utilisation enzymes to consume human milk oligosaccharides

*Akkermansia muciniphila* is adept at mucus glycans degradation and we showed in this study that *A. muciniphila* was able also to utilise human milk glycans. Thus, we investigated whether the enzymatic capacity is adapted to the different environmental conditions. Consequently, we compared the protein expression profile between *A. muciniphila* grown on human milk and grown on mucin.

**Figure 4:**
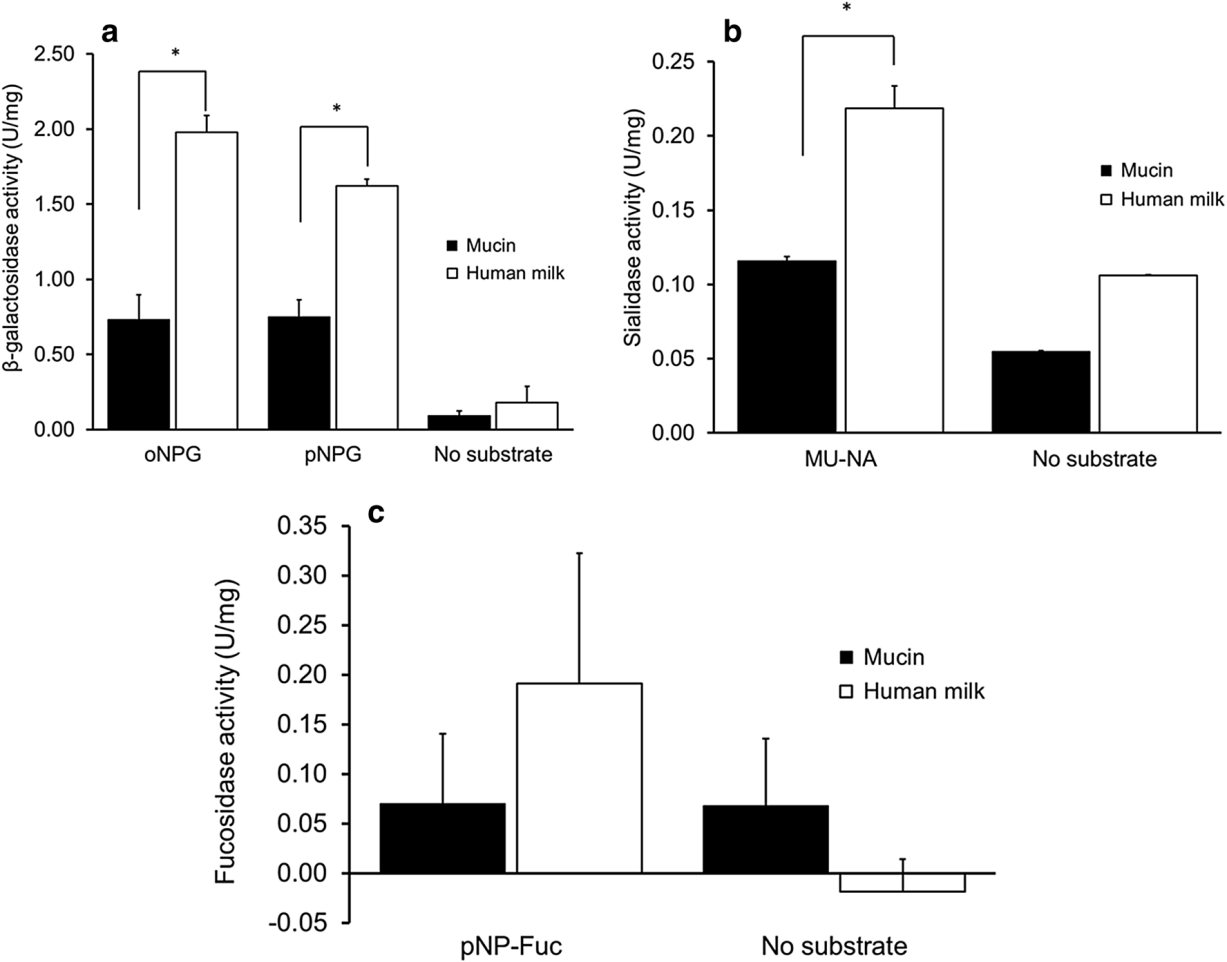
Enzymatic activity of cells lysates of *A. muciniphila.* The activity of (**a**) β-galactosidases, (**b**) sialidases and (**c**) α-fucosidases in cell lysates was tested with 2.5 mM oNPG/pNPG, 2.5 mM MU-NA and 0.5 mM pNP-Fuc, respectively. One unit (U) is the amount of enzyme that converts 1 μmole substrate per min.

First, we tested the activity of *A. muciniphila* cell lysates by measuring α-fucosidase, β-galactosidase and sialidase activities from cultures grown in either human milk or mucin. The lysates from mucin- and human milk cultures were incubated with synthetic substrates and both demonstrated fucosidase, β-galactosidase and sialidase activity. Human milk cell lysates showed significantly higher β-galactosidase and sialidase activity than in mucin lysates (Fig. 4a,b). Fucosidase activity was similar for both human milk and mucin (Fig. 4c). The cell lysates were also incubated with lactose or 2′-FL. Samples were taken at 0, 20 h and analysed by HPAEC-PAD. The lysates from *A. muciniphila* grown both on human milk and mucin showed partial degradation of lactose into its constituent monosaccharides (glucose and galactose) and partial degradation of 2′-FL into lactose, glucose, galactose and fucose (Supplementary Fig. 6a–d).

Second, a comparative proteome study identified the enzymes of *A. muciniphila* that are expressed in either human milk or mucus conditions. A total of 832 proteins were detected in both human milk and mucin. Forty-six proteins were significantly more abundant in human milk (*p*-value < 0.05), while 219 proteins more abundant in mucin condition (Supplementary Fig. 7). The rest of the proteins were expressed in similar amounts between the two conditions. We identified 108 proteins that were involved in carbohydrate metabolic process, of which the majority (67%) was expressed in similar amounts in human milk and mucin (Table 3), while 36 (33%) were significantly influenced by the environmental conditions (Supplementary Tables 5 and 6). When focusing on mucus glycan degradation enzymes, 42 out of the 61 annotated enzymes in the *A*. *muciniphila* proteome were detected in both milk and mucin conditions (Table 3). The majority (64%) was expressed in similar amounts in both conditions. Three proteins were significantly more abundant in human milk (Amuc_1755 and Amuc_1033 encoding for sulfatase activity, Amuc_0670 encoding for trypsin-like protein serine protease), and 12 proteins were more abundant in mucin condition (Amuc_1631—Carboxyl terminal protease, Amuc_1220—α-*N*-acetylglucosaminidase, Amuc_0451—Sulfatase, Amuc_0824—Glycoside Hydrolase Family 2, Amuc_1008—Glycoside Hydrolase Family 31, Amuc_1835—Exo-α-sialidase, Amuc_1182—Sulfatase, Amuc_0010—α-l-fucosidase, Amuc_0369—β-*N*-acetylhexosaminidase, Amuc_1187—α-galactosidase, Amuc_1924—β-*N*-acetylhexosaminidase, and Amuc_1815—β-*N*-acetylhexosaminidase). The majority of the glycan-degrading enzymes were found to be carbohydrate-active enzymes belonging to the glycoside hydrolases (GH) family (Table 3). Finally, human milk conditions showed a higher expression of the pili-associated protein (Amuc_1100), which has been characterised as an outer membrane protein^35^. All proteins of the pili gene cluster (Amuc_1098 – Amuc_1102), were expressed in (Amuc_1099, Amuc_1100, Amuc_1101) in higher levels in human milk, while Amuc_1098 was more abundant on mucin (*p*-value < 0.05). The results indicate that *A. muciniphila* has a highly adapted lifestyle to thrive on complex host-derived glycan structures such as the ones in human milk and the mucus layer, where similar enzymes are used by the organism to degrade either substrate.

**Table 3:**
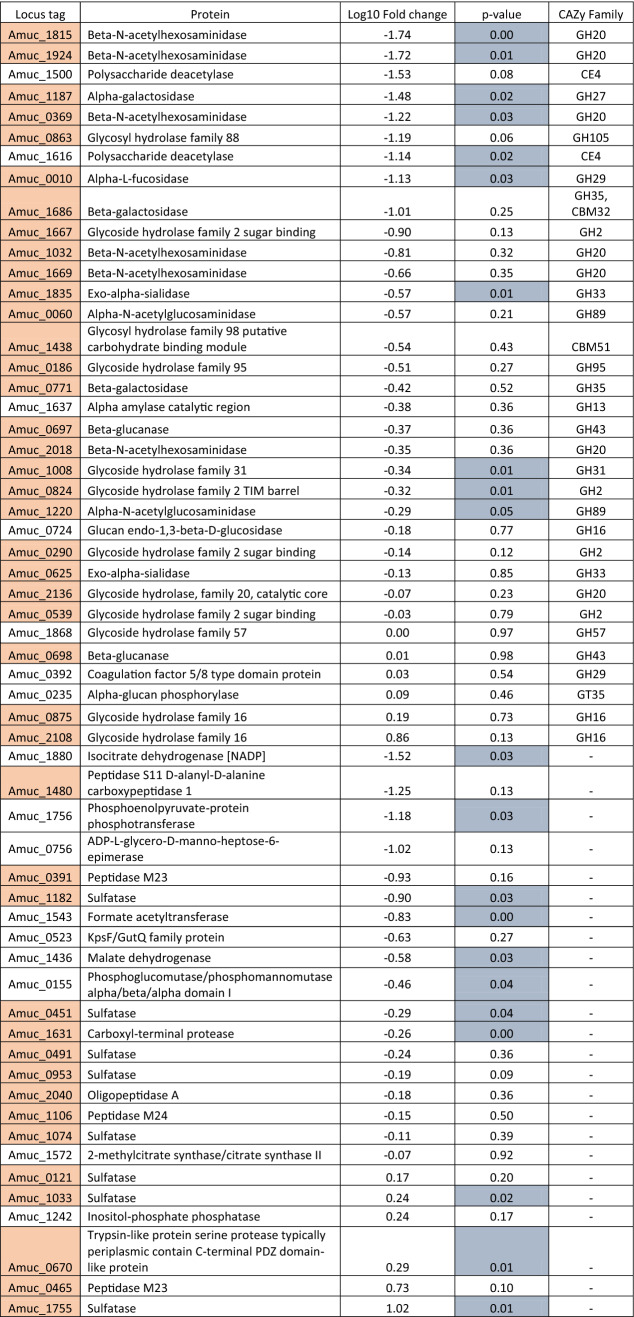
*A. muciniphila*’s saccharolytic enzymes.

Locus tags in grey indicate the enzymes that are predicted to be in mucin degradation with their corresponding CAZy Family group. Positive values (Log10 Fold change) indicate higher abundance in human milk than in mucin condition. P-values less than 0.05 are shown in with light blue colour.

## Discussion

This study demonstrates that *A. muciniphila* can grow on human milk thanks to the expression of a set of HMO degrading enzymes. Human milk fermentation by *A. muciniphila* resulted in the degradation of fucosylated and sialylated HMOs (Fig. 3). We demonstrated that *A. muciniphila* GH29 α-fucosidase (Amuc_0010) could cleave the α1-2-linked fucose to galactose. However, the high *K*_M_ value obtained during incubation with 2′-FL indicates that this substrate might not be one of the preferred substrates for Amuc_0010. In mucus, fucose is mostly found linked through α1-6 to the reducing, terminal GlcNAc^30^ in *O*-glycans, indicating that this enzyme from *A. muciniphila* might prefer mucus glycans structures over glycosidic linkages that are present in HMOs. HMO content differs per person based on the expression of certain glycosyltransferases. For instance, the presence of terminal α1-2 fucosylated HMOs requires a functional α1-2 fucosyltransferase (FUT2), encoded by the secretor gene in humans^36,37^. Interestingly, it was reported that *A. muciniphila* is more abundant in the caesarean-born infants of FUT2 secretor mothers than in the non-secretor mothers^38^. In this study, we showed that *A. muciniphila* can use fucose from human milk and basal media supplemented with 2′-FL as an energy source resulting in the production of 1,2-propanediol. This agrees with the ability of *A. muciniphila* to use fucose released from mucin. The liberation of 1,2-propanediol could lead to cross-feeding in the gut as previous studies have shown that 1,2-propanediol was utilised by other bacteria in the gastrointestinal tract^39–42^. For instance, *Eubacterium hallii* and *Lactobacillus reuteri* were able to utilise 1,2-propanediol derived from the fermentation of fucose and rhamnose to produce propionate^39,40^. In this way, the milk and mucus degrading ability of *A. muciniphila* would support microbial network formation in the early life intestine and the early life mucosal environment in the gut.

*A. muciniphila* encodes sialidases that could cleave the 3′-sialyllactose into lactose and neuraminic acid. The activity of *A. muciniphila* against sialylated mucus-glycans has been assessed before^43^. We found that the two exo-α-sialidases (Amuc_0625 and Amuc_1835) were present in our proteomics dataset. The release of α2-3 and α2-6-linked sialic acid from sialylated HMOs 3′-SL and 6′-SL^44^ are therefore considered to be the result of their activity. Sialic acid resulting from the degradation of 3′-SL could not be further utilised by *A. muciniphil*a, which could be due to the fact that it lacks the Nan cluster (NanA/K/E) that is described to be necessary for the sialic acid utilisation in other microorganisms^45^. Both human milk and mucin are rich in terminal sialyl-groups. The sialidase activity of *A. muciniphila* enables the release of sialic acid, however it seems most likely that *A. muciniphila* uses its sialidase activity to reach the sugars that are difficult to attain. At the same time, the released sialic acid could serve as substrate for community members, rather than for *A. muciniphila* itself. This implies a key role for *A. muciniphila* in the formation of a microbial network in the infant gut stimulating growth of bacteria that can use sialyl-groups and thus increasing diversity. *Bifidobacterium breve’s* growth, for example, is promoted by the sialic acid that is liberated from the degradation of sialyl-oligosaccharides in the gut by *Bifidobacterium bifidum*^46,47^*.* Additionally, *Ruminococcus gnavus* ATCC 29149 that is present in the digestive tract of humans encodes a complete Nan cluster for the sialic acid consumption^48^. It has been also reported that *Bacteroides fragilis* was able to metabolise the released sialic acid in the gut via an alternative pathway (*nanLET*)^49^. To further support the cross-feeding properties of *A. muciniphila*, it has been observed before that *A. muciniphila* is stimulated by the presence of the butyrate producer *Anaerostipes caccae* when grown on mucin*.* Indeed, *A. muciniphila* when co-cultured with *A. caccae* upregulates mucin-degrading genes that can play a role in the degradation of oligosaccharide chains consisting of monomeric sugars such as GalNAc, GlcNAc, mannose, galactose, fucose and sialic acid^50^.

In the proteome data, we further identified two β-galactosidases (Amuc_0771, Amuc_1686) of *A. muciniphila*, that showed similar expression between human milk and mucin cultures. The recombinantly-expressed protein Amuc_0771 displayed activity on lactose by releasing galactose and glucose. However, the rate of hydrolysis of the reaction was slow and we were unable to determine the kinetics of this enzyme on lactose. This finding is consistent with that of Kosciow et al. who observed that Amuc_0771 exhibited low relative hydrolytic activity against β1-4 linked galactose to glucose (lactose) and β1-4 linked galactose to *N*-acetyl-d-glucosamine (LacNAc)^51^. Guo et al., recently characterised the enzymatic activity of an *A. muciniphila* GH35 β-galactosidase, Am0874. In this study, a different tool was used for annotation *A. muciniphila’s* genes that is not in any other common genome or proteome database (ncbi.nlm.nih.gov, genome.jp/keg, uniport.org,). When we aligned the protein sequences from both β-galactosidases NCBI we found a 100% identity between Amuc_0771 and Am0874. Based on their data we can confirm that this enzyme can hydrolyse glycosidic bonds in synthetic substrates and *N*-glycans. They pointed out that the enzyme showed higher efficiency of cleaving β1-3 and β1-6 than β1-4-linked galactose. Interestingly, they found that Am0874 had lower cleaving capacity when galactose was linked to GlcNAc in β1-4-configuration compared to galactose linked in β1-3-configuration GalNAc^52^. In our results this β-galactosidase (Amuc_0771) exhibited higher hydrolysis against β1-3-linked galactose (LNT) than β1-4 configuration (lactose). The other β-galactosidase (Amuc_1686) that we studied, showed no hydrolysing capacity against LNT or lactose. The activity of Amuc_1686 has been characterised in another study where it is reported that the enzyme exhibits no activity against lactose either, but it showed preference over Galacto-*N*-biose (Galβ1- 3GalNAc)^53^. In the same study, it is mentioned that Amuc_1686 is able to cleave only the β1-3-linked galactose to GalNAc and not the β1-3-linked galactose to GlcNAc. Their findings might explain the reason why Amuc_1686 was not active against the β1-3-linked galactose to GlcNAc (LNT) in our experiments. *A. muciniphila*’s β-galactosidases are responsible for the liberation of the terminal galactose linked to milk’s glycan structures. The free galactose is consumed further by the action of a galactokinase (Amuc_0969). Surprisingly, closer analysis of the proteome data highlighted that *A. muciniphila* produced Amuc_0969 in higher amounts than Amuc_0097, indicating that galactose continued being utilised by *A. muciniphila* compared to glucose (Supplementary Table 4).

In the absence of glycoproteins from mucus *A. muciniphila* has an essential need for GlcNAc^54^*.* In case of growth on human milk GlcNAc could be released by β-acetylhexosaminidases. Wang et al., recently described two *A. muciniphila* GH20 β-acetylhexosaminidases (Am2301 and Am2446) that were able to cleave the terminal GlcNAc off the *N*- and *O*-glycans, confirming its exo-activity as glycoside hydrolases^55^. Our biochemical experiment on the purified β-acetylhexosaminidases (Amuc_0369 and Amuc_2136) revealed that both enzymes were able to hydrolyse only the terminal β1-3 linked GlcNAc to lactose (LNT2) that results to the liberation of lactose molecule and not the GlcNAc that is located inside the HMO structure (LNT).

*A. muciniphila* grown on human milk led to the production of a significant amount of succinate (~ 10 mM). *A. muciniphila* uses the succinate pathway to produce propionate in the gut, as has been described before^56^. Recently, it has been demonstrated that *A. muciniphila* is dependent on the presence of vitamin B12 as a cofactor of Methylmalonyl-CoA synthase in order to convert succinate to propionate^50,57^. *A. muciniphila* grown on human milk expresses all the necessary enzymes to successfully convert succinate to propionate (Methylmalonyl-CoA mutase; Amuc_1984, Amuc_1983 and Methylmalonyl-CoA epimerase; Amuc_0200). It is described that in human milk vitamin B12 can be tightly bound to haptocorrin, and the concentration varies depending on the diet of the mother, especially on the intake of animal products^58^. An explanation why succinate is not converted to propionate in human milk in our experiment could be that *A. muciniphila* is not efficient in using the B12 present in human milk.

*Akkermansia muciniphila* grown on human milk shows significant higher abundance of the pili-protein (Amuc_1100) (*p*-value = 0.02, 1.29-fold change)^59^. This outer membrane pili-like protein plays an important role in immune regulation and enhancement of trans-epithelial resistance. Recently, it has been demonstrated that Amuc_1100 is able to improve gut barrier and restrain the high-fat-diet-induced obesity in mice^60^. Therefore, expression of this protein in the infant gut might also contribute to immune maturation and gut health in early life.

Interestingly, the proteome analysis showed high abundance of seven sulfatases functioning as sulfate ester hydrolases. Four of these expressed sulfatases (Amuc_0451, Amuc_1033, Amuc_1182, Amuc_1755) were significantly higher expressed in human milk. It has not been reported so far that HMOs could have sulfate residues attached to their glycan structure like in *O*-glycans of mucin. However, it has been described that human milk can contain more than 100 μmol/L of sulfate esters^61^. These sulfate esters are part of the glycosaminoglycans (GAGs). GAGs are present in human milk (416 mg/L) and are highly sulfated linear polysaccharides constituted by disaccharidic units where the sugar unit is made up of an *N*-acteylhexosamine (GalNAc or GlcNAc)^62^. The most abundant GAGs found in human milk are chondroitin sulfate (CS) (231 mg/L) and heparin (Hep) (173 mg/L). Therefore, it is possible that *A. muciniphila* deploys its sulfatases for the hydrolysis of the sulfuric esters that are present in human milk glycosaminoglycans.

**Figure 5:**
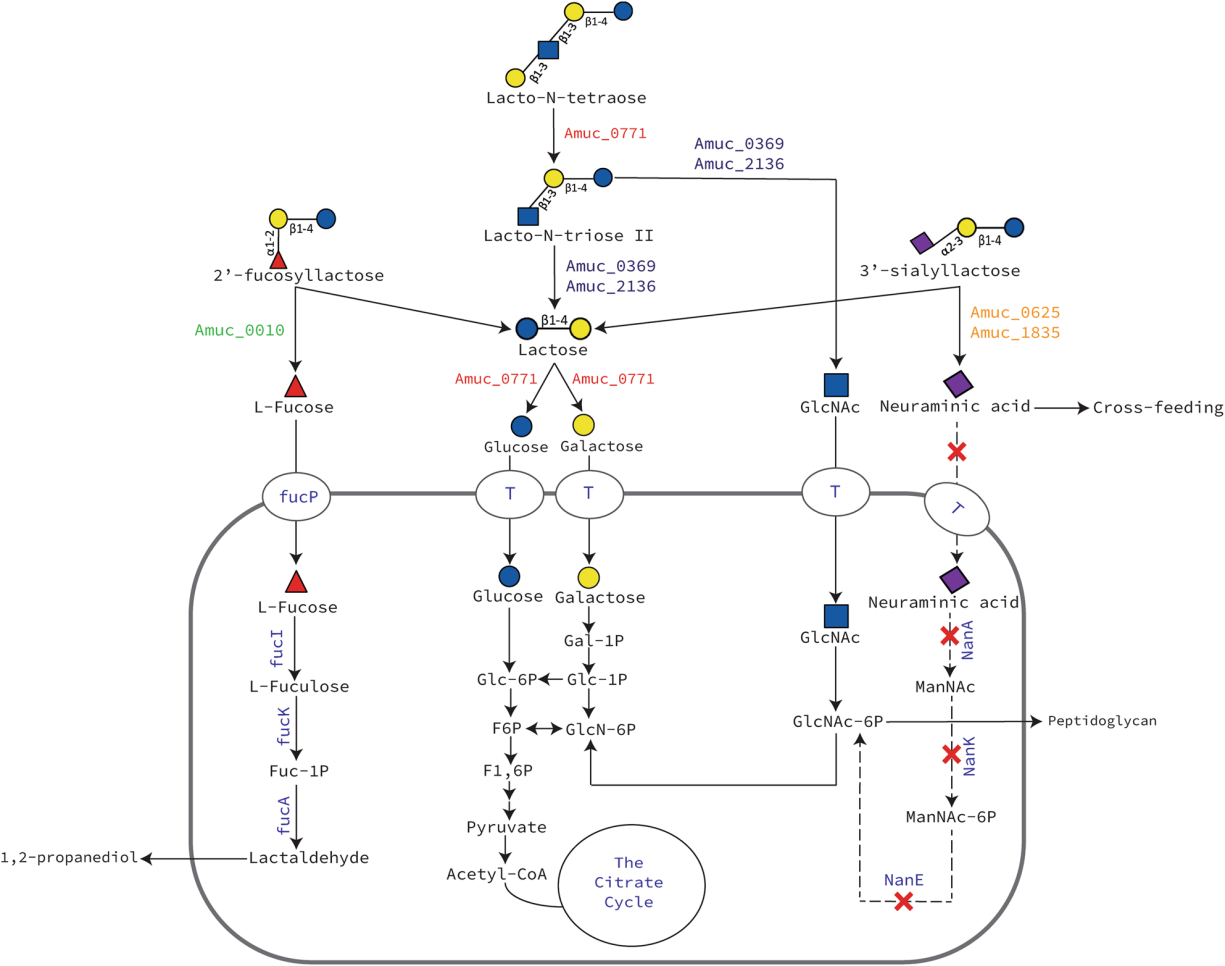
Schematic representation of the proposed pathway for the metabolism of pure 2′-fucosyllactose, 3′-sialyllactose, lacto-*N*-tetraose, lacto-*N*-triose II and lactose by *A. muciniphila.* The proteins in green colour represent α-fucosidases, in orange represent sialidases, in red represent β-galactosidases and in blue represent β-*N*-acetylhexosaminidases. *fucP* l-fucose transporter, *T* putative substrate transporter.

In this study, it is shown that *A. muciniphila* can grow on human milk and is able to degrade HMOs by using its glycan-degrading enzymes. These glycan-degrading enzymes hydrolyse the HMOs extracellularly into mono- and disaccharides and then the liberated sugars are imported in the cell by transporters^34^. These findings allow us to propose a model for the utilisation of 2′-fucosyllactose, 3′-siallylactose, lacto-*N*-tetraose, lacto-*N*-triose II and lactose by *A. muciniphila* (Fig. 5). The results of the study suggest that *A. muciniphila* is able to survive in early life environment by consuming oligosaccharides coming either from the breast milk or infant formulae. This might provide beneficial effects during the initial early life colonisation of *A. muciniphila* before it reaches its natural niche, the outer mucosal layer. Furthermore, the insufficient degradation of HMOs by *A. muciniphila* liberates simpler glycan structures and metabolites, which then become accessible to other beneficial bacteria. The milk environment also activated the expression of *A. muciniphila* outer membrane protein Amuc_1100 which could benefit the infant immune system^63,64^. Human milk carries its microbiota to the infant’s intestine facilitating early life colonisation. Development of a healthy gut microbiota from the beginning of life with glycan-degrading microbes could be associated with health in later life by guiding the development of the immune system and protecting against pathogens^65,66^. As such, *A. muciniphila* might be one of the key members of the early life guiding healthy microbiota and immune development.

## Material and methods

### Bacterial growth conditions

*Akkermansia muciniphila* Muc^T^ (ATCC BAA-835) was grown in basal medium as described previously^2^. The medium was supplemented with either hog gastric mucin (0.5% w/v Type III, Sigma-Aldrich, St. Louis, MO, USA), which was purified by ethanol precipitation as described previously^67^, human milk (10% v/v), 2′-fucosyllactose (2′-FL, 10 mM, purity > 90%) or 3′-sialyllactose (3′-SL, 10 mM, purity > 90%). The growth conditions containing HMOs (i.e. 2′-FL and 3′SL) were supplemented with 25 mM *N*-acetyl-glucosamine (GlcNAc) as nitrogen source for *A. muciniphila* (Sigma-Aldrich). The bottles containing human milk and HMOs were further supplemented with l-Proline and l-Threonine (Sigma-Aldrich) (6 g/L). All the anaerobic bottles were supplemented with 1% v/v of CaCl_2_ and vitamin mixture as described previously^68^. Human breast milk was collected under sterile conditions from a healthy donor (approximately 3–6 months post-partum) and stored at − 20 °C until use. Incubations were completed in serum bottles sealed with butyl rubber stoppers at 37 °C under anaerobic conditions provided by a gas phase of 182 kPa (1.5 atm) N_2_/CO_2_T. The bacterial growth on mucin by measuring the optical density at 600 nm and quantitative Polymerase Chain Reaction (qPCR). The optical density of the cultures of *A. muciniphila* grown in human milk could not be determined due to the high turbidity of the milk. Therefore, bacterial numbers were solely measured by qPCR.

### Quantitative real-time PCR (qPCR)

The abundance of *A. muciniphila* in human milk and mucin was determined by qPCR as described previously^10^. Cells (1 mL) were harvested at 21,000 × *g* for 15 min. DNA extractions were performed using the MasterPure™ Gram Positive DNA Purification Kit (Epicentre, Lucigen, USA). DNA concentrations were measured fluorometrically (Qubit dsDNA BR assay, Invitrogen) and adjusted to 1 ng/μL prior to use as the template in qPCR. Primers targeting the 16S rRNA gene of *A. muciniphila* (AM1 5′-CAGCACGTGAAGGTGGGGAC-3′ and AM2 5′-CCTTGCGGTTGGCTTCAGAT-3′; 327 bp ^10^) were used for quantification. A standard curve was prepared with nine standard concentrations from 10^0^ to 10^8^ gene copies/μL. qPCR was performed in triplicate with iQ SYBR green supermix (Bio-Rad, USA) in a total volume of 10 μL prepared with primers at 500 nM in 384-wells plates with the wells sealed with optical sealing tape. Amplification was performed with an iCycler (Bio-rad): one cycle of 95 °C for 10 min; 40 cycles of 95 °C for 15 s, 60° C for 20 s, and 72 °C for 30 s each; one cycle of 95 °C for 1 min; and a stepwise increase of temperature from 60 to 95 °C (at 0.5 °C per 5 s) to obtain melt curve data. Data were analysed using Bio-Rad CFX Manager 3.0. The copy number was corrected for the DNA concentration and for the number of 16S rRNA genes encoded in *A. muciniphila*’s genome.

### Human milk oligosaccharide extraction

HMOs were recovered from 1 mL aliquots of *A. muciniphila* grown in 10% v/v human milk cultures as described before^69^. An internal standard of 1,5-α-l-arabinopentaose (Megazyme, Ireland) was added, at the volume of 10 μL per sample to minimize pipetting error, to reach a final concentration of 0.01 mmol/L. The solution was diluted 1:1 with ultrapure water and centrifuged at 4,000 × *g* for 15 min at 4 °C. The supernatant was filtered through 0.2 μm syringe filter followed by subsequent centrifugation with a pre-washed ultra-filter (Amicon Ultra 0.5 Ultracel Membrane 3 kDa device, Merck Milipore, USA) at 14,000 × *g* for 1 h at room temperature. Finally, the filtrate was vortexed and stored at − 20 °C.

### Electrospray ionisation liquid chromatography mass spectrometry (LC-ESI-MS^2^) analysis

HMOs were identified and quantified with LC-ESI-MS^2^^69^. This method allows the study of distinct HMO structures more in particular their monosaccharide sequence, glycosidic linkage and the molecular conformation. Thereby the HMOs isobaric isomers such as Lacto-*N*-fucopentaose (LNFP) I, II, III and V could be distinguished as described by Mank et al.^69^. We used the latter approach with adaptations. Micro LC-ESI-MS^2^ analysis was performed on a 1,200/1,260 series HPLC stack (Agilent, Waldbronn, Germany) consisting of solvent tray, degasser, binary pump, autosampler and DAD detector coupled to a 3,200 Qtrap mass spectrometer (ABSciex, USA). After extraction of HMOs, 2.5 μL of this extract was injected into the LC–MS system. Oligosaccharides were separated by means of a 2.1 × 30 mm Hypercab porous graphitized carbon (PGC) column with a 2.1 × 10 mm PGC pre-column (Thermo Scientific, USA) using a water–methanol gradient for 30 min, and 2 min equilibration. Solvent A consisted of 0.3% ammonium hydroxide solution (28–30%, Sigma-Aldrich, St. Louis, Missouri United States) in water and solvent B of 0.3% ammonium hydroxide solution in 95% methanol (all v/v). Pre-equilibration was performed using 97.5% solvent A. The gradient started with 97.5% solvent A for 0.5 min, decreased to 60% in 12.5 min and decreased to 40% in 3 min, where it was kept for 4 min. In a next segment, solvent A decreased in 0.5 min to 2.5% where it was kept for 3 min. In 0.5 min, solvent A increased to 97.5% for re-equilibration of 6 min. Eluent flow was 400 μL/min and the columns were kept at 45 °C. The LC-effluent was infused online into the mass spectrometer and individual HMO structures were analysed semi-quantitatively by multiple reaction monitoring (MRM) in negative ion mode. Specific MRM transitions for neutral HMOs up to pentaoses and acidic HMOs up to trioses were included. The spray voltage was − 4500 V, the declustering potential and collision energy were optimized to individual compounds measured. A segmented method was used to obtain higher sensitivity. Each MRM-transition was measured for 70 ms. The instrument was calibrated with polypropylene glycol (PPG) according the instruction of the manufacturer.

### Protein extraction from *A. muciniphila* cultures

*Akkermansia muciniphila* was grown in basal medium supplemented with 0.5% purified mucin or 10% human milk (4 biological replicates). After 15 and 48 h incubation at 37 °C, cells (2 mL) were pelleted at 4,816 × *g* for 30 min at 4 °C, re-suspended in 1 mL PBS, washed twice (21,130 × *g* at 4 °C), and finally re-suspended in 500 µL lysis buffer (100 mM Tris HCl, pH 8.0, 4% (w/v) SDS, 7.7 mg/mL Dithiotreitol [DTT]). Cells were lysed by sonication (four pulses of 20 s with 30 s rest on ice) with an amplitude of 20–30% on ice using an MS-72 probe, followed by centrifugation at 21,130 × *g* for 30 min at 4 °C. Qubit® Protein Assay Kit (Life technologies, Oregon, USA) was used according to the manufacturer's instructions to determine the protein content of cell extracts. Protein samples (15 h for mucin and 48 h for human milk samples) (40 μg) were loaded on a Bolt 4–12% Bis–Tris Plus separation gel (Invitrogen, Life Technologies, USA) using the XCell Surelock Mini-Cell (Novex, Life Technologies, USA). The electrophoresis procedure was according to the manufacturer's instructions. Gels were stained overnight using QC Colloidal Coomassie Blue G250 stain (Bio-rad Laboratories, USA).

### In-gel digestion identification and relative quantification of proteins from *A. muciniphila* cell extracts

Each of the used gel lanes was cut into three slices to increase the number of identified proteins. Slices were further processed to pieces of about 1 mm^2^ and put in 1.5 mL low binding tubes (Eppendorf) prior to their reduction, alkylation, and trypsin digestion, as described previously^70^. The supernatant obtained was used for LC–MS/MS analysis. Samples were measured by nLC–MS/MS with a Proxeon EASY nLC and a LTQ-Orbitrap XL mass spectrometer as previously described^71,72^. LC–MS data analysis was performed as described previously^70,73,74^ with false discovery rates (FDRs) set to 0.01 on peptide and protein level, and additional result filtering (minimally two peptides necessary for protein identification of which at least one is unique and at least one is unmodified). Any remaining hits against the reversed database as well as all human proteins were removed. To analyse the abundance of proteins in the fractions, their label-free quantification (LFQ) intensities were compared^75^. Non-existing LFQ intensity values due to not enough quantified peptides were substituted with a value slightly lower than the lowest LFQ intensity value measured. The normal logarithm was taken from protein LFQ MS1 intensities as obtained from MaxQuant. Relative protein quantitation of sample to control was done with Perseus^76^ by applying a two sample T-test using the “LFQ intensity” columns obtained with FDR set to 0.01 and S0 set to 1. nLC-MSMS system quality was checked with PTXQC^77^ using the MaxQuant result files. The mass spectrometry proteomics data have been deposited to the ProteomeXchange Consortium via the PRIDE^78^ partner repository with the dataset identifier PXD011357. All the *A. muciniphila* proteins differentially expressed between milk and mucin conditions are listed in Supplementary Tables 5 and 6.

### Cloning, expression and purification of selected *A. muciniphila* glycoside hydrolases

The selected proteins coded by the genes with locus tags Amuc_0010 (57.51 kDa), Amuc_0369 (72.02 kDa), Amuc_0771 (70.53 kDa), Amuc_1686 (85.71 kDa), and Amuc_2136 (81.59 kDa) without their signal peptides were cloned into the pCDF-1b vector, introducing a 6xHis-tag at the C-terminus. The genes of interest and the plasmid backbone were amplified by PCR with gene-specific primers, using Q5 DNA polymerase (New England BioLabs, USA) (Supplementary Table 1). The amplified genes and vector were being assembled with NEBuilder HiFi DNA Assembly Master Mix (NEB BioLabs, USA) according to manufacturer’s instructions. *E. coli* DH5-aplha competent cells were transformed using 5 μL of assembly reaction and sequences were verified by DNA sequencing by Eurofins (Ebersberg, Germany). *E. coli* BL21 Rosetta competent cells were transformed with the recombinant plasmid harbouring the genes of interest. The recombinant cells were grown to an OD_600_ between 0.5 and 0.6 in 250 mL Lysogeny–Broth (LB) supplemented with 50 μg/mL spectinomycin and 25 μg/mL chloramphenicol. Then, they were induced with 1 mM isopropyl β-D-1-thiogalactopyranoside (IPTG) at 22 °C for 16 h. The cells were harvested by centrifugation at 6,000 × *g* for 20 min and re-suspended in 15 mL of lysis buffer. Cells were lysed by sonication on ice (1 s pulse with 2 s rest for 10 min, amplitude of 20–25%) using a MS-72 microtip (Bandelin, Germany). The lysate was cleared by centrifugation at 120,000 × *g* for 1 h at 4 °C. The proteins were His-tag purified using Ni^2+^-nitrilotriacetate (Ni–NTA) agarose affinity columns (500 µL bed volume, Qiagen) equilibrated with binding buffer. Samples were loaded, followed by 2 mL of binding buffer and 2 × 500 µL plus 1 mL of elution buffer. Purity of the enzymes was checked by SDS-Page (4–10% polyacrylamide gel) (Supplementary Fig. 2) and pure samples were concentrated using 50-kDa MWCO Amicon Ultra-15 Centrifugal Filter Units (Merck, Germany) with storage buffer. Protein content was measured using the Qubit Protein Assay (manufacturer’s protocol) and fluorometer (DeNovix, USA). pCDF-1b empty vector was cloned, expressed and purified as it is described above. The empty vector was used as negative control in all the enzymatic assays.

### Activity assays and kinetics

The purified enzymes were incubated with different substrates in McIlvaine buffer solution adjusted to the optimum pH of each enzyme at 37 °C. To quantify α-fucosidase, β-galactosidase and β-acetylhexosaminidase activity on synthetic substrates, 4-nitrophenyl-a-l-fucopyranoside (pNP-α-l-Fuc), 4-nitrophenyl-b-d-galactopyranoside (pNPG) and 4-nitrophenyl 2-acetamido-2-deoxy-b-d-glucopyranoside (GlcNAc-β-pNP) were used respectively. The enzymes were incubated with substrate concentrations ranging from 0 to 5 mM in McIlvaine buffer. At intervals of 2.5 min, samples were taken and added to 2.5 × volume of glycine buffer (pH 9.6) to stop the reaction. Absorbance was monitored at 405 nm and quantified using a standard curve of 4-Nitrophenol (pNP). To determine the optimal pH of each enzyme, the assay was carried out in McIlvaine buffer with pH values ranging from 5.0 to 8.2 and a substrate concentration of ~ 3 times the K_M_ as determined at the initial pH. Next, the aforementioned assays with 0–5 mM substrate were repeated at the optimal pH. For incubations of β-galactosidases and hexosaminidases with lactose and LNT, the assays were performed in a similar way, but the products were monitored by HPAEC-PAD. The sugars were separated by HPAEC with 10 mM NaOH at 0.5 mL/min on a CarboPac PA20 protected with a guard column and detected using PAD on a Dionex ICS5000 system (Thermo Scientific). The column was cleaned for 10 min with 200 mM NaOH and re-equilibrated with 10 mM NaOH. Standards of galactose and glucose in different concentrations were used to quantify the results. For incubations of the α-fucosidase with 2′-FL, reactions were performed in a similar way, but the fucose release was measured directly (without adding glycine buffer first) by α-l-fucose kit (Megazyme, Ireland) based on a fucose dehydrogenase-coupled method^79^. Reactions without enzyme and with only the purified plasmid incubated in both synthetic and human milk derived sugars were served as negative controls. Kinetic data of the enzymes were obtained from triplicate experiments, and the kinetic parameters were calculated by fitting the initial raw data to the Michaelis–Menten equation using linear regression analysis, and the error bars (SD).

### Cell lysate activity

Enzymatic activity of *A. muciniphila* cell lysates was tested with colorimetric substrates as described previously^80^. The following substrate/enzyme combinations were used: 4-nitrophenyl-α-l-fucopyranoside/fucosidase (pNP-Fuc), 4-nitrophenyl-β-d-galactopyranoside (pNPG) and 2-nitrophenyl-β-d-galactopyranoside (ONPG)/β-galactosidase, and 2′-(4-methylumbelliferyl)-α-d-*N*-acetylneuraminic acid (MU-NA)/sialidase. Reactions were performed in a final volume 20 µL, using 0.5–2.5 mM substrate and ~ 1 µg/mL enzyme in 0.05 M citrate buffer pH 6.0 (fucosidase, sialidase) or 0.25 M phosphate buffer, pH 7.0 (β-galactosidase). After 1 h of incubation at 37 °C, reactions were stopped by adding 50 µL 0.5 glycine buffer, pH 9.6. Absorbance was read at 405 nm (pNPG and pNP-Fuc) or 420 nm (oNPG) and fluorescence intensity was measured at λ_ex_ 390 nm, λ_em_ 460 nm on a 96-well plate reader (Biotek, USA). Activity is expressed in units (U), in which one unit is the amount of enzyme that converts 1 µmole substrate per minute. To test fucosidase and galactosidase activity of cell lysates in 2′-FL and lactose, ~ 10 µg/mL of each enzyme was incubated with 1 mM lactose and 1 mM of 2′-FL in 0.05 mM citrate buffer, pH 6.0 in a final volume of 1 mL, shaking at 300 rpm. Reactions of aliquots at different time points were stopped by adding 2.5 × the volume of 0.5 M glycine buffer, pH 9.6. The reaction was monitored by HPAEC-PAD as it was described previously.

### High-performance liquid chromatography

For fermentation product analysis, samples were obtained at different time points of the incubation period. Crotonate was used as the internal standard and the external standards were lactate, formate, acetate, propionate, butyrate, succinate, 1,2-propanediol, lactose, *N-*acetyl-glucosamine (GlcNAc), *N-*acetyl-galactosamine (GalNAc), 2′-fucosyllactose (2′-FL), 3′-sialyllactose (3′-SL), glucose, galactose, fucose and sialic acid. Substrate conversion and product formation were measured with Thermo Scientific Spectrasystem high-performance liquid chromatography (HPLC) system equipped with a Varina Metacarb 67H 300 × 6.5 mm column kept at 45 °C and running 0.0005 mM sulfuric acid as eluent. The eluent had a flow of 0.5 mL/min and metabolites were detected by determining the refractive index (RI-150) and identified by using standards of pure compounds as described previously^81^.

### Statistical analysis

Statistics were performed using **s**tudent’s t-test and corrected for multiple testing using False Discovery Rate (FDR). Data are presented as mean ± standard deviation (SD), unless stated otherwise. *P*-values below 0.05 were considered significant.

### Ethical consideration

The usage and analysis of human milk samples described in this study were performed with written consent by the donor.

## Supplementary information

Supplementary Information.
