# Supplementary material for: Akkermansia muciniphila uses human milk oligosaccharides to thrive in the early life conditions in vitro
Source: Sci Rep. 2020 Aug 31;10:14330. doi: 10.1038/s41598-020-71113-8 (PMC7459334; doi:10.1038/s41598-020-71113-8)
Supplement: Supplementary file 1 — Supplementary Information. [file 41598_2020_71113_MOESM1_ESM.pdf]

**Supplemental file**

**for**

# ***AKKERMANSIA MUCINIPHILA* USES HUMAN MILK OLIGOSACCHARIDES TO THRIVE IN THE EARLY CONDITIONS *IN VITRO***

Ioannis Kostopoulos[1], Janneke Elzinga[1], Noora Ottman[1], Jay T. Klievink[4], Bernadet Blijenberg[3], Steven Aalvink[1], Sjef Boeren[2], Marko Mank[3], Jan Knol[1,3], Willem M. de Vos[1,4], and Clara Belzer[1,*]

[1]Laboratory of Microbiology, Wageningen University, Stippeneng 4, 6708 WE Wageningen, The Netherlands, [2]Laboratory of Biochemistry, Wageningen University, Stippeneng 4, 6708 WE Wageningen, The Netherlands, [3]Danone Nutricia Research, Uppsalalaan 12, 3584 CT Utrecht, The Netherlands, [4]Research Program Human Microbiome, Faculty of Medicine, University of Helsinki, P.O.Box 66, FI-00014, Helsinki, Finland.

[*]Corresponding author: Clara Belzer (clara.belzer@wur.nl)

**Supplementary Table 1:** The primers used for transformation of Amuc_0369, Amuc_2136, Amuc_0010, Amuc_0771, Amuc_1686 and vector into *E. coli* BL21 Rosetta, including 6xHis-Tag on C-terminus. His-tags are given in bold.

| Protein name | Protein | Forward primer (5'-3') | Reverse primer (5'-3') |
|---|---|---|---|
| β-hexosaminidase | Amuc_0369 | GGATCCCAATTGGGAGCTCATGGAACAAATCATCCCGAAACC | TTA**GTGGTGATGGTG**ATGATGTGGCGTTTCAAAATACACTGTC |
| β-hexosaminidase | Amuc_2136 | GGATCCCAATTGGGAGCTCATGAAGCAGATTGCGGATTCC | TTA**GTGGTGATGGTG**ATGATGCAACGGCTGTACGTTCAT |
| α-fucosidase | Amuc_0010 | GGATCCCAATTGGGAGCTCATGCAGTCCGCCACTAAAATC | CTA**GTGGTGATGGTG**ATGATGTTTGCTCAGTTTGATGACGG |
| β-galactosidase | Amuc_0771 | GGATCCCAATTGGGAGCTCATGGCATTGGCATGGGC | TTA**GTGGTGATGGTG**ATGATGCCACTGGTTGTCCAGGTTTTT |
| β-galactosidase | Amuc_1686 | GGATCCCAATTGGGAGCTCATGCCCATGCCTTTGC | TTA**GTGGTGATGGTG**ATGATGCTTGGCAGGCTTGAACG |
| Backbone (pcdf-1b) | - | **CATCATCACCATCACCAC**TAGCTCGAGTCTGGTAAAGAAACCG | GAGCTCCCAATTGGGATCC |
| | - | **CATCATCACCATCACCAC**TAACTCGAGTCTGGTAAAGAAACCG | |
| | - | **CATCATCACCATCACCAC**TGACTCGAGTCTGGTAAAGAAACCG | |

**Supplementary Table 2:** Fermentation products and sugar utilisation of *A. muciniphila* grown on human milk and mucin.

| Mucin | | | | | | | |
|---|---|---|---|---|---|---|---|
| Time (hours) | Acetate (mM) | Propionate (mM) | 1,2-propanediol (mM) | Succinate (mM) | Lactose (mM) | Glucose (mM) | Galactose (mM) |
| 0 | 0.00 ± 0.00 | 0.00 ± 0.00 | 0.00 ± 0.00 | 0.00 ± 0.00 | 0.03 ± 0.06 | 0.04 ± 0.10 | 0.04 ± 0.10 |
| 4 | 0.94 ± 1.06 | 0.27 ± 0.30 | 0.00 ± 0.00 | 0.24 ± 0.12 | 0.00 ± 0.00 | 0.13 ± 0.20 | 0.13 ± 0.20 |
| 15 | 16.53 ± 4.55 | 11.72 ± 2.70 | 0.45 ± 0.60 | 0.74 ± 0.58 | 0.00 ± 0.00 | 0.08 ± 0.13 | 0.08 ± 0.13 |
| 18 | 17.17 ± 3.65 | 12.98 ± 1.93 | 0.40 ± 0.48 | 0.18 ± 0.42 | 0.00 ± 0.00 | 0.00 ± 0.00 | 0.00 ± 0.00 |
| 24 | 17.21 ± 4.76 | 12.66 ± 2.26 | 0.50 ± 0.80 | 0.15 ± 0.38 | 0.02 ± 0.06 | 0.14 ± 0.22 | 0.14 ± 0.22 |
| 48 | 17.05 ± 4.14 | 12.55 ± 1.91 | 0.72 ± 1.00 | 0.14 ± 0.34 | 0.00 ± 0.00 | 0.09 ± 0.15 | 0.09 ± 0.15 |
| 72 | 16.82 ± 4.32 | 12.56 ± 1.99 | 0.57 ± 0.81 | 0.00 ± 0.00 | 0.00 ± 0.00 | 0.13 ± 0.21 | 0.13 ± 0.21 |
| 96 | 17.33 ± 4.53 | 12.75 ± 2.01 | 0.58 ± 0.80 | 0.00 ± 0.00 | 0.00 ± 0.00 | 0.11 ± 0.18 | 0.11 ± 0.18 |
| 120 | 17.55 ± 3.78 | 13.08 ± 1.72 | 0.59 ± 0.79 | 0.00 ± 0.00 | 0.00 ± 0.00 | 0.07 ± 0.11 | 0.07 ± 0.11 |
| Human Milk | | | | | | | |
| Time (hours) | Acetate (mM) | Propionate (mM) | 1,2-propanediol (mM) | Succinate (mM) | Lactose (mM) | Glucose (mM) | Galactose (mM) |
| 0 | 0.00 ± 0.00 | 0.00 ± 0.00 | 0.90 ± 1.43 | 0.00 ± 0.00 | 21.85 ± 3.75 | 0.78 ± 0.48 | 0.01 ± 0.03 |
| 4 | 0.01 ± 0.03 | 0.00 ± 0.00 | 0.00 ± 0.00 | 0.00 ± 0.00 | 20.69 ± 3.31 | 0.83 ± 0.76 | 0.07 ± 0.16 |
| 15 | 1.00 ± 0.68 | 1.61 ± 1.71 | 0.00 ± 0.00 | 0.16 ± 0.40 | 20.78 ± 1.37 | 0.79 ± 0.31 | 0.40 ± 0.13 |
| 18 | 1.46 ± 1.02 | 1.42 ± 1.00 | 0.00 ± 0.00 | 0.39 ± 0.61 | 20.23 ± 2.21 | 0.88 ± 0.25 | 0.54 ± 0.18 |
| 24 | 2.09 ± 1.49 | 2.08 ± 1.23 | 0.00 ± 0.00 | 0.56 ± 0.60 | 18.58 ± 2.82 | 1.34 ± 0.58 | 0.94 ± 0.48 |
| 48 | 4.29 ± 2.44 | 4.39 ± 2.65 | 0.00 ± 0.00 | 2.05 ± 1.41 | 14.13 ± 1.40 | 7.31 ± 2.93 | 4.44 ± 2.44 |
| 72 | 9.23 ± 2.19 | 9.55 ± 1.79 | 0.00 ± 0.00 | 8.14 ± 2.00 | 5.52 ± 1.22 | 11.00 ± 1.76 | 6.41 ± 1.30 |
| 96 | 12.15 ± 2.15 | 12.28 ± 1.79 | 0.03 ± 0.06 | 9.87 ± 0.96 | 0.59 ± 0.69 | 11.87 ± 2.43 | 7.10 ± 1.50 |
| 120 | 12.05 ± 2.28 | 13.10 ± 2.40 | 0.04 ± 0.11 | 10.51 ± 0.77 | 0.30 ± 0.25 | 13.19 ± 2.59 | 8.12 ± 1.40 |

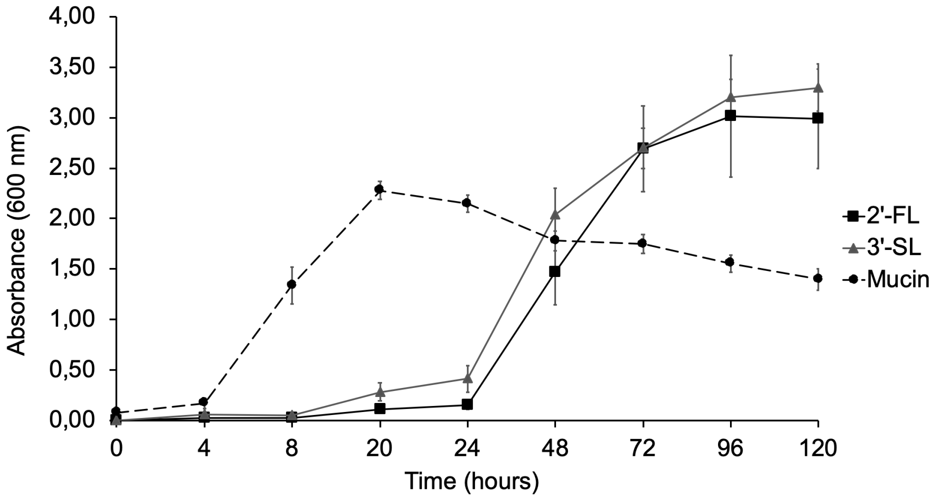


**Supplementary Figure 1:** Growth curve of *A. muciniphila* grown in mucin (black dotted line), 2'-FL (black line), 3'-SL (grey line) measured by optical density at 600 nm.

**Supplementary Table 3:** a) Carbon balance of *Akkermansia muciniphila* on HMOs and GlcNAc after 48 hours of fermentation, b) Production of sugars and metabolites of *A. muciniphila* grown in 2'-FL (top) and 3'-SL (bottom).

a)

| | | Substrates | | Products | | | | | | | | | | | Carbon recovery (%) | |
|---|---|---|---|---|---|---|---|---|---|---|---|---|---|---|---|---|
| HMO | Time point (h) | 2'-fucosyllactose | GlcNAc | 2'-fucosyllactose | GlcNac | GalNac | Fucose | Lactose | 1,2-propanediol | Glucose | Galactose | Acetate | Propionate | Lactate | Average | SD |
| 2'-fucosyllactose | 48 | 81.58 | 89.26 | 81.58 | 89.26 | 2.79 | 9.24 | 31.42 | 2.05 | 4.55 | 4.88 | 31.75 | 28.33 | 0.00 | 86.58 | 3.45 |
| HMO | Time point (h) | 3'-sialyllactose | GlcNAc | 3'-sialyllactose | GlcNac | GalNac | Fucose | Lactose | Neuraminic Acid | Glucose | Galactose | Acetate | Propionate | Lactate | Average | SD |
| 3'-sialyllactose | 48 | 172.66 | 60.82 | 172.66 | 60.82 | 2.90 | 0.20 | 8.59 | 47.76 | 2.48 | 2.81 | 42.82 | 35.02 | 0.00 | 82.79 | 9.10 |

b)

| 2'-FL | | | | | | | | | | | |
|---|---|---|---|---|---|---|---|---|---|---|---|
| Time (hours) | Acetate (mM) | Propionate (mM) | 1,2-propanediol (mM) | GalNAc (mM) | GlcNAc (mM) | Succinate (mM) | Galactose (mM) | Glucose (mM) | Lactose (mM) | 2'-Fucosyllactose (mM) | Fucose (mM) |
| 0 | 0.00 ± 0.00 | 0.00 ± 0.00 | 0.00 ± 0.00 | 0.29 ± 0.04 | 22.27 ± 1.76 | 0.00 ± 0.00 | 0.00 ± 0.00 | 0.00 ± 0.00 | 0.00 ± 0.00 | 8.29 ± 0.33 | 0.00 ± 0.00 |
| 4 | 0.00 ± 0.00 | 0.00 ± 0.00 | 0.00 ± 0.00 | 0.63 ± 0.14 | 23.30 ± 1.86 | 0.00 ± 0.00 | 0.43 ± 0.61 | 0.00 ± 0.00 | 0.00 ± 0.00 | 8.81 ± 0.42 | 0.00 ± 0.00 |
| 8 | 0.22 ± 0.29 | 0.00 ± 0.00 | 0.00 ± 0.00 | 0.76 ± 0.14 | 23.67 ± 2.20 | 0.00 ± 0.00 | 0.57 ± 0.81 | 0.00 ± 0.00 | 0.00 ± 0.00 | 8.95 ± 0.52 | 0.00 ± 0.00 |
| 20 | 2.06 ± 0.08 | 3.19 ± 0.21 | 0.00 ± 0.00 | 0.46 ± 0.32 | 20.73 ± 1.75 | 0.00 ± 0.00 | 0.02 ± 0.03 | 0.00 ± 0.00 | 0.00 ± 0.00 | 7.92 ± 0.51 | 0.04 ± 0.05 |
| 24 | 4.50 ± 3.32 | 3.20 ± 1.05 | 0.00 ± 0.00 | 0.47 ± 0.22 | 18.79 ± 3.95 | 0.00 ± 0.00 | 0.33 ± 0.47 | 0.00 ± 0.00 | 0.00 ± 0.00 | 7.24 ± 1.61 | 0.64 ± 0.63 |
| 48 | 15.88 ± 3.52 | 9.44 ± 2.15 | 0.68 ± 0.26 | 0.35 ± 0.10 | 11.16 ± 0.36 | 0.00 ± 0.00 | 0.81 ± 0.26 | 0.76 ± 0.16 | 2.62 ± 0.72 | 4.53 ± 0.06 | 1.54 ± 0.18 |
| 72 | 26.27 ± 6.89 | 18.01 ± 5.04 | 3.22 ± 1.33 | 0.26 ± 0.10 | 1.00 ± 0.21 | 0.00 ± 0.00 | 0.38 ± 0.18 | 0.49 ± 0.05 | 3.00 ± 0.30 | 0.61 ± 0.72 | 0.33 ± 0.17 |
| 96 | 33.46 ± 1.38 | 24.78 ± 0.59 | 5.24 ± 0.28 | 0.00 ± 0.00 | 0.07 ± 0.10 | 0.00 ± 0.00 | 0.55 ± 0.38 | 0.58 ± 0.32 | 1.96 ± 0.69 | 0.09 ± 0.09 | 0.45 ± 0.48 |
| 120 | 35.82 ± 0.34 | 27.52 ± 0.63 | 5.96 ± 0.42 | 0.00 ± 0.00 | 0.00 ± 0.00 | 0.00 ± 0.00 | 0.46 ± 0.49 | 0.46 ± 0.40 | 0.77 ± 0.97 | 0.10 ± 0.06 | 0.51 ± 0.35 |

| 3'-SL | | | | | | | | | | |
|---|---|---|---|---|---|---|---|---|---|---|
| Time (hours) | Acetate (mM) | Propionate (mM) | GalNAc (mM) | GlcNAc (mM) | Succinate (mM) | Galactose (mM) | Glucose (mM) | Lactose (mM) | 3'-Sialyllactose (mM) | Neuraminic acid (mM) |
| 0 | 0.00 ± 0.00 | 0.00 ± 0.00 | 0.38 ± 0.40 | 24.31 ± 2.16 | 0.00 ± 0.00 | 0.02 ± 0.03 | 0.00 ± 0.00 | 0.11 ± 0.04 | 11.09 ± 2.14 | 0.38 ± 0.66 |
| 4 | 0.00 ± 0.00 | 0.00 ± 0.00 | 0.31 ± 0.00 | 24.66 ± 0.04 | 0.00 ± 1.71 | 0.12 ± 0.00 | 0.00 ± 0.19 | 0.10 ± 0.00 | 11.37 ± 2.24 | 0.44 ± 0.76 |
| 8 | 0.70 ± 0.07 | 0.00 ± 0.07 | 0.32 ± 0.00 | 23.77 ± 0.04 | 0.00 ± 1.99 | 0.10 ± 0.00 | 0.00 ± 0.18 | 0.07 ± 0.00 | 10.96 ± 2.00 | 0.41 ± 0.70 |
| 20 | 3.77 ± 0.95 | 2.58 ± 0.95 | 0.32 ± 0.00 | 21.33 ± 0.02 | 0.00 ± 1.26 | 0.00 ± 0.00 | 0.00 ± 0.00 | 0.26 ± 0.00 | 10.70 ± 2.05 | 1.82 ± 0.48 |
| 24 | 4.68 ± 1.39 | 2.65 ± 1.39 | 0.10 ± 0.00 | 21.10 ± 0.18 | 0.00 ± 1.63 | 0.00 ± 0.00 | 0.00 ± 0.00 | 0.20 ± 0.00 | 10.79 ± 1.87 | 1.46 ± 0.47 |
| 48 | 21.41 ± 3.62 | 11.67 ± 3.62 | 0.36 ± 0.00 | 7.60 ± 0.03 | 0.00 ± 1.71 | 0.47 ± 0.00 | 0.41 ± 0.16 | 0.72 ± 0.06 | 7.51 ± 1.85 | 4.34 ± 2.60 |
| 72 | 31.44 ± 2.20 | 19.35 ± 2.20 | 0.52 ± 0.00 | 2.10 ± 0.14 | 0.00 ± 0.45 | 1.17 ± 0.00 | 0.97 ± 0.22 | 3.39 ± 0.13 | 1.11 ± 0.98 | 12.73 ± 2.06 |
| 96 | 33.96 ± 3.41 | 22.79 ± 3.41 | 0.49 ± 0.00 | 0.54 ± 0.52 | 0.00 ± 0.54 | 1.26 ± 0.00 | 0.84 ± 0.29 | 2.18 ± 0.23 | 0.00 ± 0.00 | 11.89 ± 3.03 |
| 120 | 36.55 ± 2.82 | 25.67 ± 2.82 | 0.00 ± 0.00 | 0.00 ± 0.00 | 0.00 ± 0.00 | 0.51 ± 0.00 | 0.20 ± 0.30 | 0.57 ± 0.34 | 0.00 ± 0.00 | 10.97 ± 2.54 |

**Supplementary Table 4: Abundance of *A. muciniphila* enzymes involved in carbohydrate metabolism with their corresponding KEGG identifier (KO ID).** The average of Log10 transformed LFQ values is shown. Colouring is based on abundance from the most abundant (red) to medium abundant (orange) to least abundant (green).

| Locus tag | Protein | Average_LFQ_Human Milk | KO ID | Pathway |
|---|---|---|---|---|
| Amuc_1210 | Phosphoenolpyruvate carboxykinase [GTP] | 9.03 | ko00010;ko00020;ko00620 | Glycolysis/Gluconeogenesis; Citrate Cycle (TCA cycle); Pyruvate metabolism |
| Amuc_1417 | Glyceraldehyde-3-phosphate dehydrogenase, type I | 8.95 | ko00010 | Glycolysis/Gluconeogenesis |
| Amuc_1832 | L-fucose isomerase | 8.61 | ko00051 | Fructose and mannose metabolism |
| Amuc_1630 | Serine hydroxymethyltransferase | 8.47 | | |
| Amuc_0309 | 2,3-bisphosphoglycerate-independent phosphoglycerate mutase | 8.09 | ko00010;ko00680 | Glycolysis/Gluconeogenesis;Methane metabolism |
| Amuc_1418 | Phosphoglycerate kinase | 8.07 | ko00010 | Glycolysis/Gluconeogenesis |
| Amuc_1975 | Glucose-6-phosphate isomerase | 7.95 | ko00010;ko00030;ko00500;ko00520 | Glycolysis/Gluconeogenesis; Pentose phosphate pathway; Starch and sucrose metabolism; Amino sugar and nucleotide sugar metabolism |
| Amuc_0721 | Fructose-bisphosphate aldolase, class II | 7.85 | ko00010;ko00030;ko00051;ko00680 | Glycolysis/Gluconeogenesis; Pentose phosphate pathway; Fructose and mannose metabolism; Methane metabolism |
| Amuc_1413 | Capsular exopolysaccharide family | 7.76 | | |
| Amuc_1184 | Enolase 2 | 7.71 | ko00010;ko00680;ko03018 | Glycolysis/Gluconeogenesis;Methane metabolism;RNA degradation |
| Amuc_1125 | UDP-glucose 4-epimerase | 7.56 | ko00052;ko00520 | Galactose metabolism; Amino sugar and nucleotide sugar metabolism |
| Amuc_0844 | Enolase 1 | 7.30 | ko00010;ko00680;ko03018 | Glycolysis/Gluconeogenesis;Methane metabolism;RNA degradation |
| Amuc_1481 | 6-phosphofructokinase | 7.15 | ko00010;ko00030;ko00051;ko00052;ko00680 | Glycolysis/Gluconeogenesis; Pentose phosphate pathway; Fructose and mannose metabolism; Amino sugar and nucleotide sugar metabolism; Methane metabolism |
| Amuc_0660 | UDP-N-acetylmuramate/alanine ligase | 7.08 | | |
| Amuc_1471 | Ribose-phosphate pyrophosphokinase | 7.01 | ko00030;ko00230 | Pentose phosphate pathway; Purine metabolism |
| Amuc_0653 | UDP-N-acetylmuramyl-tripeptide synthetase | 7.00 | ko00300;ko00550 | Lysine biosynthesis; Peptidoglycan biosynthesis |
| Amuc_0654 | UDP-N-acetylmuramoyl-tripeptide--D-alanyl-D-alanine ligase | 6.95 | ko00300;ko00550 | Lysine biosynthesis; Peptidoglycan biosynthesis |
| Amuc_1388 | 4-hydroxy-3-methylbut-2-en-1-yl diphosphate synthase | 6.89 | ko00900 | Terpenoid backbone biosynthesis |
| Amuc_1822 | Glucosamine-6-phosphate isomerase | 6.79 | ko00520 | Amino sugar and nucleotide sugar metabolism |
| Amuc_1741 | UTP--glucose-1-phosphate uridylyltransferase | 6.72 | ko00040;ko00052;ko00500;ko00520 | Pentose and glucuronate interconversions; Galactose metabolism; Starch and sucrose metabolism; Amino sugar and nucleotide sugar metabolism |
| Amuc_0431 | Pyruvate kinase | 6.71 | ko00010;ko00230;ko00620 | Glycolysis/Gluconeogenesis; Purine metabolism; Pyruvate metabolism |
| Amuc_0562 | Triosephosphate isomerase | 6.62 | ko00010;ko00051;ko00562 | Glycolysis/Gluconeogenesis; Fructose and mannose metabolism; Inositol phosphate metabolism |
| Amuc_0772 | Phosphoglycerate mutase 1 family | 6.60 | | |
| Amuc_1249 | GDP-mannose 4,6-dehydratase | 6.59 | ko00051;ko00520 | Fructose and mannose metabolism; Amino sugar and nucleotide sugar metabolism |
| Amuc_0969 | Galactokinase | 6.54 | ko00052;ko00520 | Galactose metabolism; Amino sugar and nucleotide sugar metabolism |
| Amuc_0210 | 6-phosphofructokinase 1 | 6.09 | ko00010;ko00030;ko00051;ko00052;ko00680 | Glycolysis/Gluconeogenesis; Pentose phosphate pathway; Fructose and mannose metabolism; Amino sugar and nucleotide sugar metabolism; Methane metabolism |
| Amuc_1309 | Aldose 1-epimerase | 6.05 | ko00010 | Glycolysis/Gluconeogenesis |
| Amuc_0661 | D-alanine--D-alanine ligase | 6.04 | ko00473;ko00550 | D-Alanine metabolism; Peptidoglycan biosynthesis |
| Amuc_0676 | 2-dehydro-3-deoxyphosphooctonate aldolase | 5.98 | ko00540 | Lipopolysaccharide biosynthesis |
| Amuc_1248 | NAD-dependent epimerase/dehydratase | 5.94 | ko00051;ko00520 | Fructose and mannose metabolism; Amino sugar and nucleotide sugar metabolism |
| Amuc_1671 | Phosphofructokinase | 5.92 | ko00010;ko00030;ko00051;ko00052;ko00680 | Glycolysis/Gluconeogenesis; Pentose phosphate pathway; Fructose and mannose metabolism; Amino sugar and nucleotide sugar metabolism; Methane metabolism |
| Amuc_0664 | Cell wall hydrolase/autolysin | 5.89 | | |
| Amuc_1192 | Mannose-1-phosphate guanylyltransferase (GDP) | 5.71 | ko00051;ko00520 | Fructose and mannose metabolism; Amino sugar and nucleotide sugar metabolism |
| Amuc_1435 | Nucleotide sugar dehydrogenase | 5.63 | ko00040;ko00053;ko00500;ko00520 | Pentose and glucuronate interconversions; Galactose metabolism; Starch and sucrose metabolism; Amino sugar and nucleotide sugar metabolism |
| Amuc_0097 | ROK family protein | 5.58 | ko00010;ko00052;ko00500;ko00520;ko00521 | Glycolysis/Gluconeogenesis; Galactose metabolism; Starch and sucrose metabolism; Amino sugar and nucleotide sugar metabolism; Streptomycin biosynthesis |
| Amuc_0550 | 3D domain protein | 5.52 | | |
| Amuc_1895 | UDP-N-acetylglucosamine 1-carboxyvinyltransferase | 5.50 | ko00520;ko00550 | Amino sugar and nucleotide sugar metabolism; Peptidoglycan biosynthesis |
| Amuc_1830 | L-fuculokinase | 5.47 | ko00051 | Fructose and mannose metabolism |
| Amuc_0169 | 3-deoxy-manno-octulosonate cytidylyltransferase | 5.00 | ko00540 | Lipopolysaccharide biosynthesis |
| Amuc_0208 | Glucose-1-phosphate thymidylyltransferase | 5.00 | ko00521;ko00523 | Streptomycin biosynthesis; Polyketide sugar unit biosynthesis |
| Amuc_0414 | Phosphoheptose isomerase | 5.00 | ko00540 | Lipopolysaccharide biosynthesis |
| Amuc_0656 | UDP-N-acetylmuramoylalanine--D-glutamate ligase | 5.00 | ko00471;ko00550 | D-Glutamine and D-glutamate metabolism; Peptidoglycan biosynthesis |
| Amuc_0681 | UDP-N-acetylmuramate | 5.00 | | |
| Amuc_0795 | Glycerol-3-phosphate dehydrogenase [NAD(P)+] | 5.00 | ko00564 | Glycerophospholipid metabolism |
| Amuc_1211 | N-acetylmuramoyl-L-alanine amidase family 2 | 5.00 | | |
| Amuc_1541 | Lipid A biosynthesis acyltransferase | 5.00 | ko00540 | Lipopolysaccharide biosynthesis |
| Amuc_1646 | 4-hydroxy-3-methylbut-2-enyl diphosphate reductase | 5.00 | ko00900 | Terpenoid backbone biosynthesis |

**Supplementary Table 5:** All the differentially expressed *A. muciniphila* proteins found in Human milk compared to mucin cultures.

| Gene names | Protein names | Log10 fold_change | *p*-value |
|---|---|---|---|
| Amuc_0037 | Amino acid permease-associated region | 0.65 | 0.00 |
| Amuc_0172 | Putative uncharacterized protein | 0.22 | 0.00 |
| Amuc_0201 | Carboxyl transferase | 0.27 | 0.00 |
| Amuc_0335 | ABC transporter related | 0.51 | 0.01 |
| Amuc_0372 | Glutamate decarboxylase | 0.67 | 0.00 |
| Amuc_0419 | General substrate transporter | 0.45 | 0.00 |
| Amuc_0468 | Transcriptional regulator, DeoR family | 0.42 | 0.03 |
| Amuc_0541 | Ribonuclease, Rne/Rng family | 0.28 | 0.00 |
| Amuc_0543 | Putative uncharacterized protein | 0.24 | 0.00 |
| Amuc_0544 | Tetratricopeptide TPR_2 repeat protein | 0.14 | 0.01 |
| Amuc_0627 | Putative uncharacterized protein | 0.41 | 0.01 |
| Amuc_0670 | Trypsin-like protein serine protease typically periplasmic contain C-terminal PDZ domain-like protein | 0.29 | 0.01 |
| Amuc_0687 | Outer membrane autotransporter barrel domain protein | 0.89 | 0.00 |
| Amuc_0705 | D-isomer specific 2-hydroxyacid dehydrogenase NAD-binding | 0.23 | 0.02 |
| Amuc_0714 | UPF0365 protein | 0.36 | 0.04 |
| Amuc_0738 | GMP synthase, large subunit | 0.27 | 0.01 |
| Amuc_0836 | ATP-dependent chaperone ClpB | 0.24 | 0.00 |
| Amuc_0841 | Argininosuccinate lyase | 0.25 | 0.02 |
| Amuc_0876 | Heavy metal translocating P-type ATPase | 0.18 | 0.04 |
| Amuc_0955 | Ribosome-binding factor A | 0.47 | 0.02 |
| Amuc_0970 | SSS sodium solute transporter superfamily | 0.23 | 0.01 |
| Amuc_1033 | Sulfatase | 0.24 | 0.02 |
| Amuc_1091 | Cytochrome-c peroxidase | 0.28 | 0.01 |
| Amuc_1099 | Putative uncharacterized protein | 0.46 | 0.03 |
| Amuc_1100 | Putative uncharacterized protein | 0.37 | 0.02 |
| Amuc_1101 | Cell division protein FtsA | 0.43 | 0.00 |
| Amuc_1184 | Enolase 2 | 0.62 | 0.00 |
| Amuc_1225 | Inorganic diphosphatase | 0.66 | 0.00 |
| Amuc_1310 | 17 kDa surface antigen | 0.62 | 0.00 |
| Amuc_1408 | 60 kDa chaperonin | 0.28 | 0.00 |
| Amuc_1443 | Putative uncharacterized protein | 0.39 | 0.00 |
| Amuc_1525 | Putative uncharacterized protein | 0.68 | 0.00 |
| Amuc_1584 | Type II secretion system protein | 0.64 | 0.00 |
| Amuc_1586 | Type II secretion system protein E | 0.59 | 0.00 |
| Amuc_1656 | Putative uncharacterized protein | 0.52 | 0.00 |
| Amuc_1689 | Dihydrolipoyl dehydrogenase | 0.44 | 0.03 |
| Amuc_1695 | Cytochrome bd ubiquinol oxidase subunit I | 0.76 | 0.00 |
| Amuc_1751 | Glycoside hydrolase family 13 domain protein/ 1,4 -alpha-glucan branching enzyme | 0.61 | 0.04 |
| Amuc_1755 | Sulfatase | 1.02 | 0.01 |
| Amuc_1776 | TPR repeat-containing protein | 0.93 | 0.00 |
| Amuc_1782 | Glutamyl-tRNA(Gln) amidotransferase subunit A | 0.15 | 0.01 |
| Amuc_1869 | Glycosyl transferase group 1 | 0.74 | 0.00 |
| Amuc_2001 | Putative lipoprotein | 0.36 | 0.02 |
| Amuc_2034 | Methylmalonyl-CoA carboxytransferase | 0.12 | 0.02 |
| Amuc_2043 | RND efflux system, outer membrane lipoprotein, NodT family | 0.45 | 0.01 |
| Amuc_2127 | Carbohydrate-selective porin OprB | 0.53 | 0.00 |

**Supplementary Table 6:** Top 10 abundant *A. muciniphila* proteins found in human milk compared to 10 most significantly abundant in mucin cultures.

| Environmental condition | Protein name | KO ID | Proteins | Kegg Brite Functional Category | Log10 Fold change | p-value |
|---|---|---|---|---|---|---|
| **Human Milk** | Amuc_1755 | x | Sulfatase | x | 1.015 | 0.01 |
| | Amuc_1776 | x | TPR repeat-containing protein | x | 0.934 | 0.00 |
| | Amuc_0687 | x | Outer membrane autotransporter barrel domain protein | x | 0.892 | 0.00 |
| | Amuc_1695 | K00425 | Cytochrome bd ubiquinol oxidase subunit I | Energy metabolism | 0.758 | 0.00 |
| | Amuc_1869 | x | Glycosyl transferase group 1 | x | 0.737 | 0.00 |
| | Amuc_1525 | x | Putative uncharacterised protein | x | 0.681 | 0.00 |
| | Amuc_0372 | K01580 | Glutamate decarboxylase | Carbohydrate metabolism, amino acid metabolism | 0.668 | 0.00 |
| | Amuc_1225 | K15986 | Inorganic diphosphatase | Energy metabolism | 0.655 | 0.00 |
| | Amuc_0037 | K20265 | Amino acid permease | Membrane transport | 0.648 | 0.00 |
| | Amuc_1584 | K02653 | Type II secretion system protein | Membrane transport | 0.645 | 0.00 |
| **Mucin** | Amuc_0557 | K02954 | 30S ribosomal protein S14 | Translation | -2.886 | 0.00 |
| | Amuc_0243 | x | Putative uncharacterized protein | x | -2.441 | 0.00 |
| | Amuc_1476 | K02990 | 30S ribosomal protein S6 | Translation | -2.389 | 0.00 |
| | Amuc_2106 | K00029 | Malate dehydrogenase (Oxaloacetate-decarboxylating) (NADP(+)) | Carbohydrate metabolism | -2.254 | 0.00 |
| | Amuc_1430 | K02909 | 50S ribosomal protein L31 | Translation | -2.183 | 0.03 |
| | Amuc_1068 | K00789 | S-adenosylmethionine synthase | Amino acid metabolism | -2.182 | 0.00 |
| | Amuc_1059 | K07239 | Acriflavin resistance protein | Cellular process and signalling | -2.174 | 0.00 |
| | Amuc_1254 | K03217 | Membrane protein oxaA | Folding, sorting and degradation, membrane transport, translation | -2.072 | 0.02 |
| | Amuc_0070 | K01867 | Tryptophanyl-tRNA synthetase | Translation, amino acid metabolism | -2.045 | 0.00 |
| | Amuc_2099 | x | Putative uncharacterized protein | x | -2.044 | 0.00 |

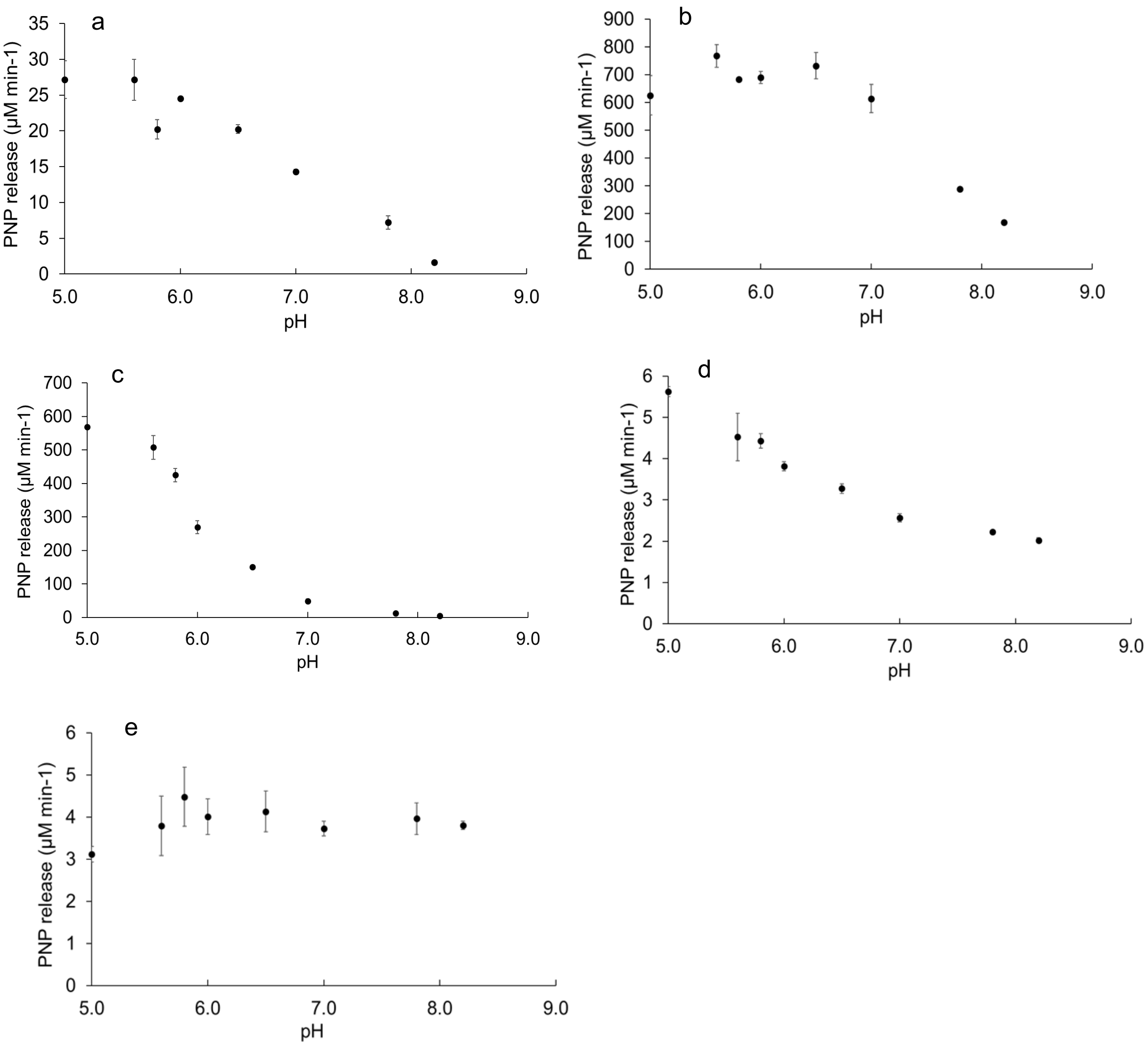


**Supplementary Figure 2: pH dependent of *A. muciniphila* GHs using pNP substrates.** a) Amuc_0010 and PNP-fucose, b) Amuc_0369 and GlcNAc-PNP, c) Amuc_2136 and GlcNAc-PNP, d) Amuc_0771 and PNPG, e) Amuc_1686 and PNPG

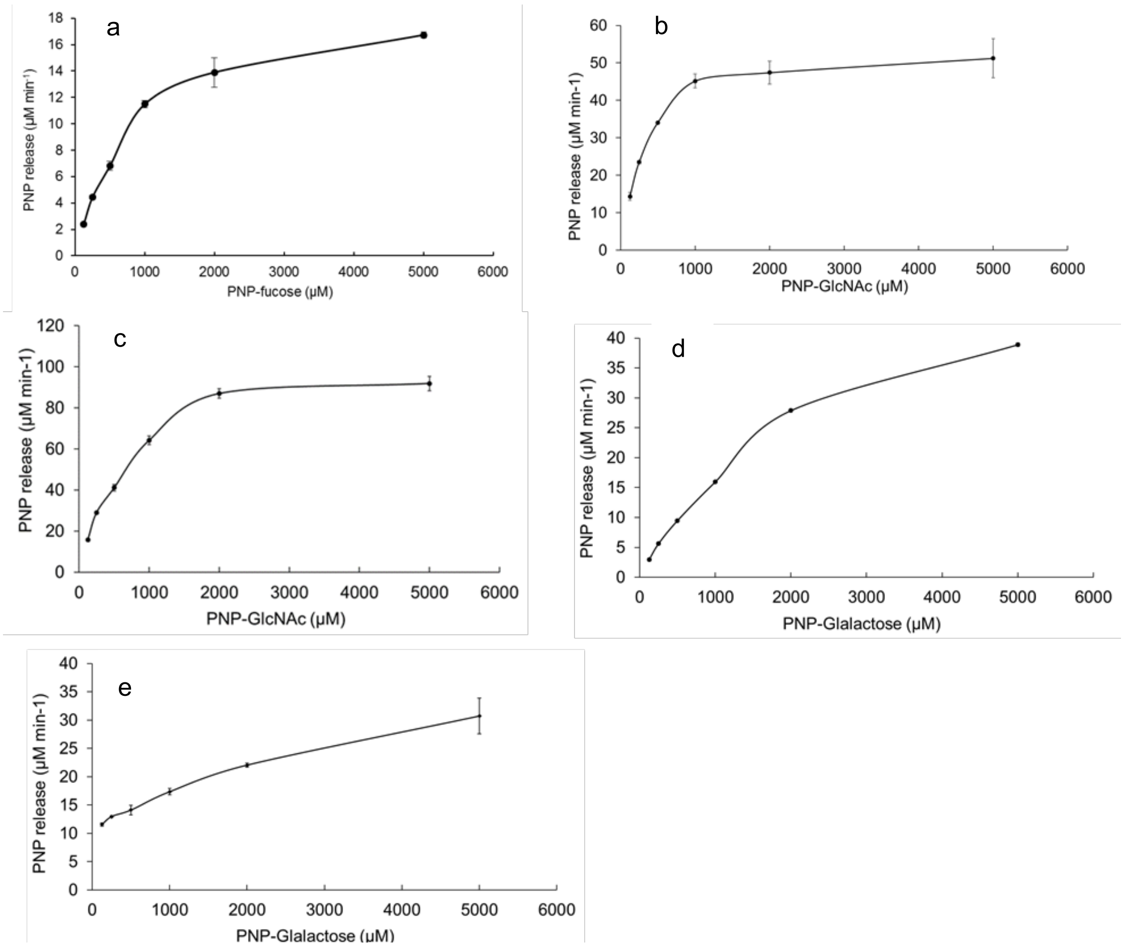


**Supplementary Figure 3: Kinetic analysis of *A. muciniphila* GHs against PNP substrates.** a) Amuc_0010 and PNP-fucose, b) Amuc_0369 and GlcNAc-PNP, c) Amuc_2136 and GlcNAc-PNP, d) Amuc_0771 and PNPG, e) Amuc_1686 and PNPG

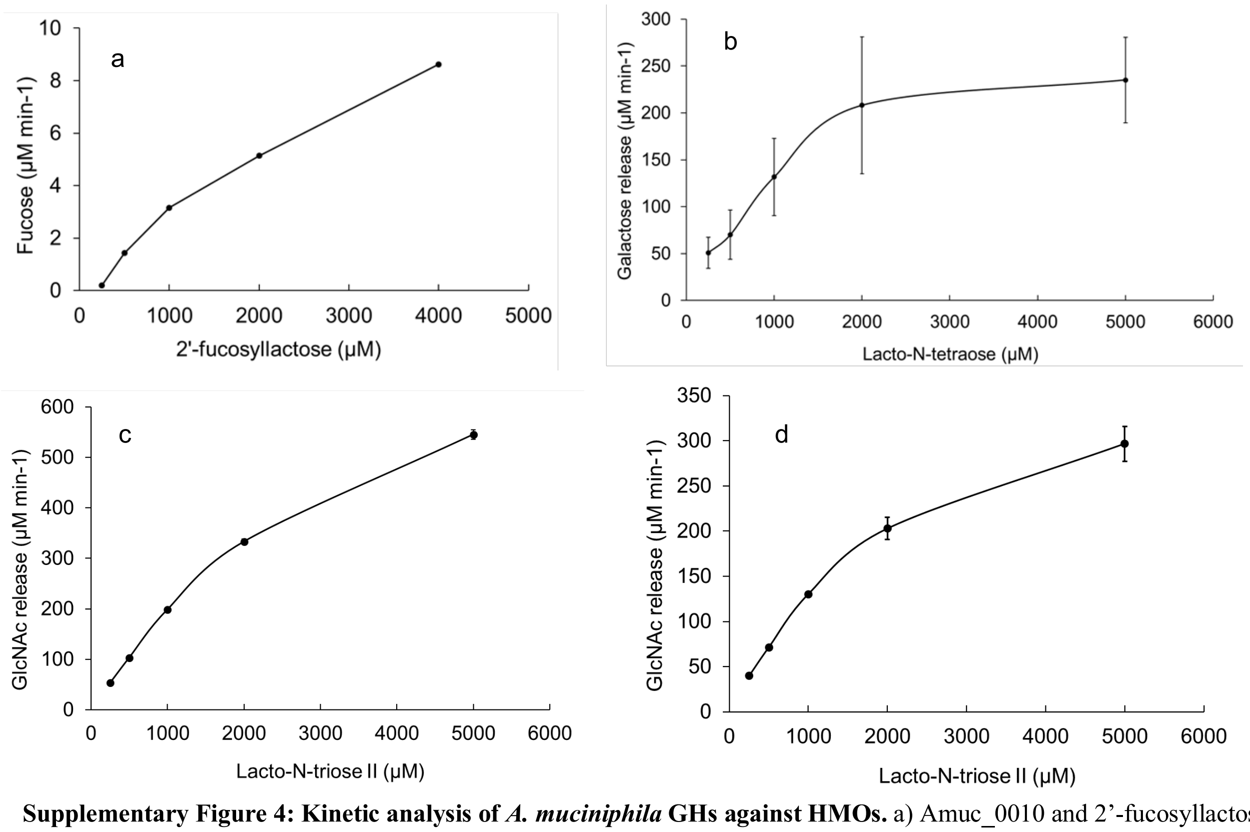


**Supplementary Figure 4: Kinetic analysis of *A. muciniphila* GHs against HMOs.** a) Amuc_0010 and 2'-fucosyllactose, b) Amuc_0771 and Lacto-N-tetraose, c) Amuc_0369 and Lacto-N-triose II, d) Amuc_2136 and Lacto-N-triose II.

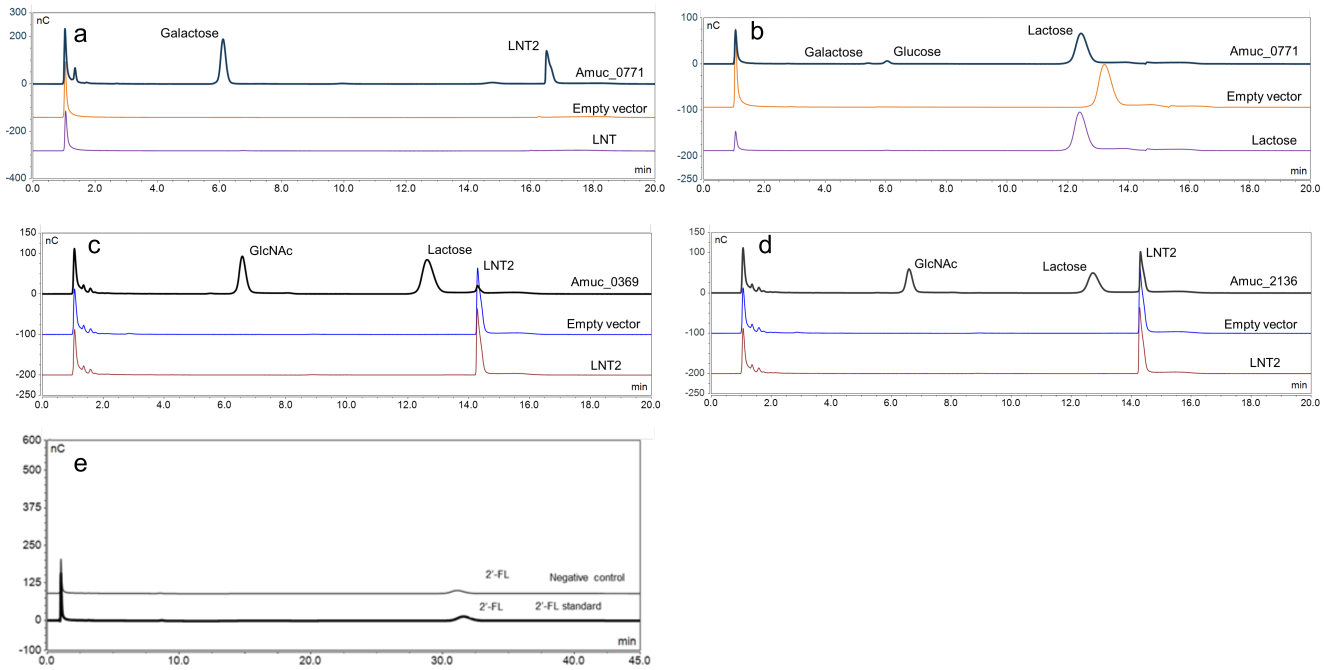


**Supplementary Figure 5: Purified *E. coli* BL21 Rosetta strain (Empty vector) specificity against HMOs and lactose.** a) Lacto-N-tetraose, b) lactose, c) and d) Lacto-N-triose II, e) 2'-fucosyllactose

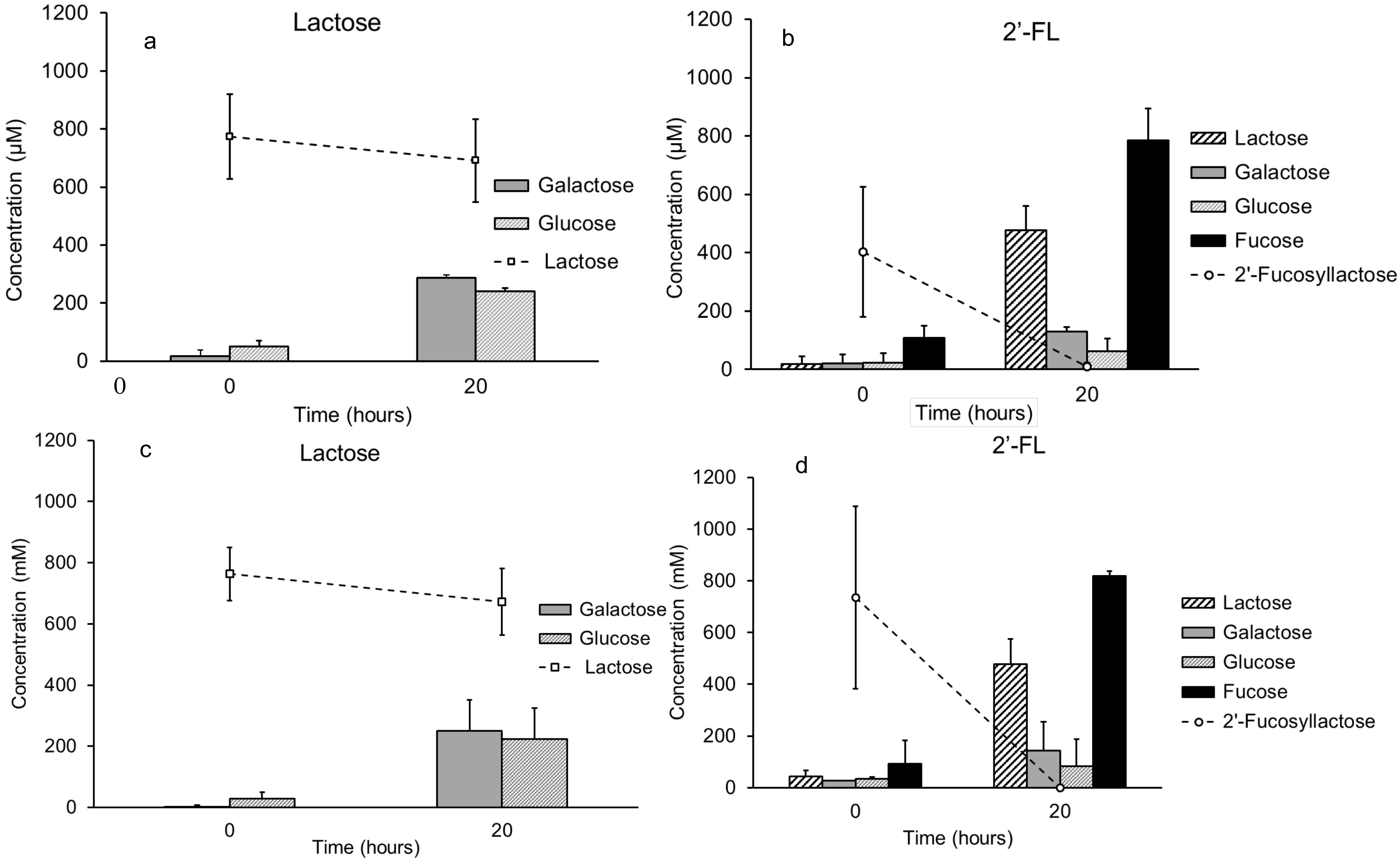


**Supplementary Figure 6: Enzymatic activity of cell lysates of *A. muciniphila* against lactose and 2'-FL.** a,b) human milk cell lysate and c,d) mucin cell lysate.

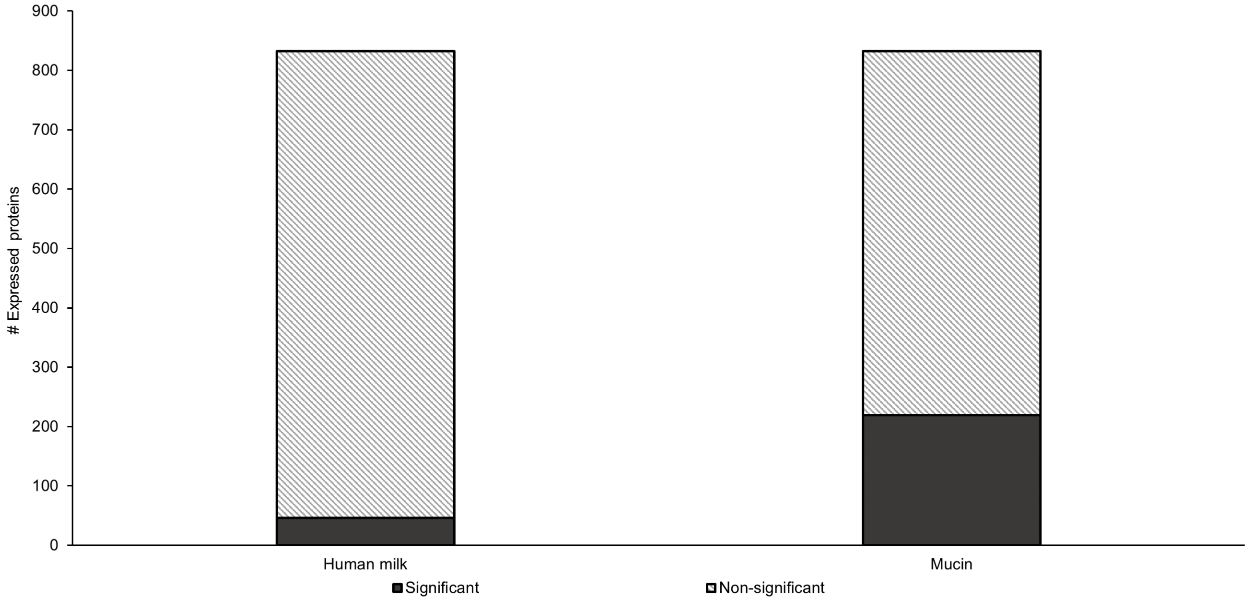


**Supplementary Figure 7:** Indication of the amount of significant and non-significant proteins expressed by *A. muciniphila* either in human milk or mucin condition.

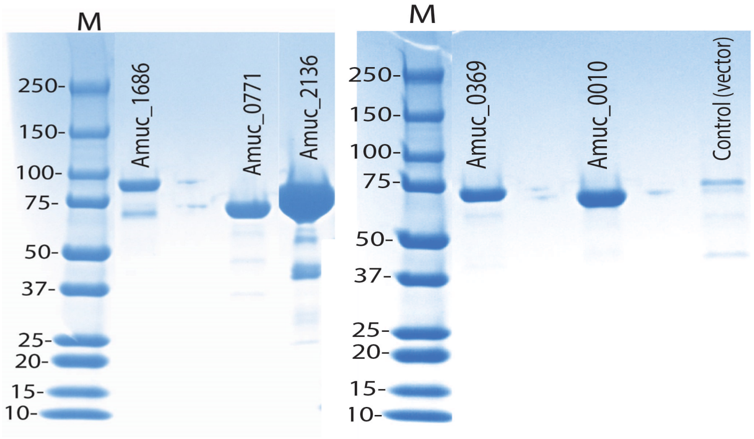


**Supplementary Figure 8: SDS-PAGE analysis of the purified recombinant *A. muciniphila* GHs.** M- protein marker.
